# Supplementary material for: Simultaneous nanocatalytic surface activation of pollutants and oxidants for highly efficient water decontamination
Source: Nat Commun. 2022 May 30;13:3005. doi: 10.1038/s41467-022-30560-9 (PMC9151758; doi:10.1038/s41467-022-30560-9)
Supplement: Supplementary file 1 — Supplementary Information [file 41467_2022_30560_MOESM1_ESM.pdf]

Supplementary Information

Simultaneous Nanocatalytic Surface Activation of Pollutants and Oxidants for Highly Efficient Water Decontamination

Ying-Jie Zhang[1]†, Gui-Xiang Huang[1]†, Lea R. Winter[2], Jie-Jie Chen[1], Lili Tian[3], Shu-Chuan Mei[1], Ze Zhang[4], Fei Chen[1], Zhi-Yan Guo[1], Rong Ji[3], Ye-Zi You[4], Wen-Wei Li[1], Xian-Wei Liu[1], Han-Qing Yu[1]*, Menachem Elimelech[2]*

[1]Department of Environmental Science and Engineering, University of Science and Technology of China, Hefei 230026, China

[2]Department of Chemical and Environmental Engineering, Yale University, New Haven, CT 06520, USA

[3]School of the Environment, Nanjing University, Nanjing 210023, China

[4]Department of Polymer Science and Engineering, University of Science and Technology of China, Hefei 230026, China

Contents:

Supplementary Materials and Methods

Supplementary Figures 1-55

Supplementary Tables 1 to 6

Supplementary Notes 1 to 6

Supplementary References

Supplementary Methods

Quantification of Pollutants and Oxidants

In the batch experiments, one milliliter of the suspension was withdrawn and quenched with ascorbic acid solution (AA:PMS = 30:1 in molar concentration) at given time intervals once the reaction was initiated. Then, the quenched samples were centrifuged to separate the solid and liquid. The supernatants were collected and analyzed for the contaminant concentration using ultra-high performance liquid chromatography (UHPLC) (1290 Infinity, Agilent, Inc. USA) with a C18 column and an acetonitrile/water (containing 0.1% formic acid) mixture as the mobile phase. For PhOH measurements, the solvent ratio of the mixture was 20:80 (v:v), and the detection wavelength was 270 nm. For BPA, the ratio was 40:60, and the wavelength was 273 nm. For 4-CP, the ratio was 45:55, and the wavelength was 280 nm. For 2,6-M-PhOH, the ratio was 20:80, and the wavelength was 273 nm. For MOP, the ratio was 20:80, and the wavelength was 273 nm. For MHB, the ratio was 25:75, and the wavelength was 255 nm. For AAP, the ratio was 5:95, and the wavelength was 242 nm. For AN, the ratio was 25:75 (without adding 0.1% formic acid), and wavelength was 258 nm. For SA, the ratio was 5:95, and wavelength was 280 nm.

The oxidant (i.e., PMS or PDS) concentration was analyzed using KI spectrophotometry[1]. Briefly, 2 mL of suspension was withdrawn at given time intervals and filtered with a 0.22-μm polytetrafluoroethylene (PTFE) syringe filter immediately. Then, 1 mL of the filtrate was mixed with 1 mL of KI solution (100 g/L, containing 5 g/L $NaHCO_3$). After reacting for 30 min, the mixture was analyzed by a UV-visible absorption spectrometer at 396 nm.

Total Organic Carbon (TOC) Measurement

In the low-concentration system, 10 mL of suspension was withdrawn from the reaction system at given time intervals and filtered with a 0.22-μm PTFE syringe filter

immediately. Then, the TOC concentration of the filtrate was analyzed using a TOC analyzer (Muti N/C 2100, Analytik Jena AG, Germany).

Chemical Oxygen Demand (COD) Measurement

In the high-concentration system, the COD of the suspension (containing solution and catalyst), bulk solution, and catalyst powder were each measured. For the suspension, 2 mL was withdrawn at given time intervals and used directly as the sample for immediate COD measurement. For the bulk solution, 3 mL of the suspension was withdrawn at given time intervals and filtered with a 0.22-μm PTFE syringe filter immediately, and then 2 mL of the filtrate was used as the sample. For the catalyst powder, 100 mL of the suspension was filtered with the 0.22-μm filter membrane, and then the powder was collected and dispersed into the same amount of pure water using ultrasound. Subsequently, 2 mL of the dispersion was used as the sample.

The above samples were mixed with the COD reagents comprising 1 mL of $K_2Cr_2O_7$ solution (0.16 M, in 10 v/v% H_2SO_4/H_2O), 0.5 mL of $HgSO_4$ solution (100 g/L, in 10 v/v% H_2SO_4/H_2O), and 4 mL of Ag_2SO_4 solution (10 g/L, in concentrated H_2SO_4). The mixtures were heated at 165 ºC for 30 min and then cooled to ambient temperature. The obtained clear solutions were analyzed using a UV-visible spectrophotometer at 440 nm.

During the oxidative coupling/polymerization reaction, theoretically one PMS can consume two electrons; for the complete oxidation of PhOH, one PhOH donates 28 electrons: 1PhOH + oxidants → $6CO_2 + 3H_2O + 28e^-$. Therefore, with the fixed molar ratio of dosed PMS to PhOH as 2:1, 4/28 of COD_0 (i.e., the initial COD) should be consumed (or oxidized) by PMS, so 6/7 of COD_0 should remain after the full reaction (in the solution and catalyst).

During the whole reaction with the PMS to PhOH molar ratio of 2:1, because there is no pollutant mineralization, the TOC of the suspension (containing solution and catalyst) did not change; that is, TOC = TOC_0 (the initial TOC).

If we define $TOC_0 = COD_0$, then the remaining COD is equal to 6/7 TOC (in the solution and catalyst) after the full reaction with persulfate (because 4/28 of COD_0 was consumed by the PMS oxidation, whereas the TOC remained unchanged). Since the TOC on the catalyst could not be directly measured by TOC analyzer, it can be estimated by the conversion of COD. Since all the products were transferred to the catalyst surface, the TOC on the catalyst could be obtained as 7/6 COD.

In the same way, after the full reaction, the remaining TOC = 10/9 COD for 2,6-M-PhOH, the remaining TOC = 18/17 COD for BPA, and the remaining TOC = 13/11 COD for 4-CP.

^{14}C Labeling Experiments

Initially, 10 μL of ^{14}C-PhOH (containing 0.17 μg ^{14}C-PhOH in chemical mass) dissolved in methanol was added into a reactor and volatilized naturally for 5 min to remove the solvent. Then, 20 mL of PhOH suspension (containing 250 mg/L PhOH solution and 1.0 g/L Co_3O_4 catalyst) was added to the reactor. After a 15-min ultrasonic dispersion and a 15-min stirring, PMS was added to initiate the reaction. At given time intervals, 1 mL of reaction suspension was withdrawn and quenched with ascorbic acid solution (AA:PMS = 30:1 in molar concentration). The radioactivity of both the suspension (containing solution and catalyst) and the solution (filtered with a 0.22-μm PTFE syringe filter) were measured using a liquid scintillation counter (LSC) with a Beckman LS6500 scintillation counter (Beckman Coulter, USA) and a Gold Star scintillation cocktail (Meridian Biotechnologies Ltd., UK). The ^{14}C-PhOH served as a radioactive indicator and did not disturb the reaction of PhOH (concentration ratio of 0.0085:250 for ^{14}C-PhOH:standard-PhOH).

Structure Characterizations

The crystal structure and morphology of the catalysts were characterized using X-ray

powder diffraction (XRD, TTR-III diffractometer, Rigaku Co., Japan) with Cu *K*α radiation (λ = 1.5418 Å), scanning electron microscopy (SEM, Phenom ProX, Phenom-World Co., Netherlands), and transmission electron microscopy (TEM, H7650, Hitachi Co., Japan). The high angle annular dark field scanning transmission electron microscopy (HAADF-STEM) images and corresponding energy dispersive spectroscopic (EDS) mapping analyses were performed on a JEOL JEM0ARF200F TEM/STEM with a spherical aberration corrector (Talos F200X, FEI Co., USA). The chemical compositions and the valence states of constituent elements were analyzed using X-ray photoelectron spectroscopy (XPS, ESCALAB250, Thermo Fisher Inc., USA) with an Al *K*α radiation source, and the binding energy was calibrated with the C1s peak at 284.8 eV. The surface properties of the catalysts were characterized using Fourier transform infrared spectroscopy (FTIR, Vertex 70, Bruker Co., Germany). The reaction products accumulated on the catalyst were analyzed using thermal gravimetric analysis-Fourier transform infrared spectroscopy (TGA-FTIR, TL-9000, Perkin Elmer Co., USA)

Scaling-Up Experiment and Product Identification

To collect and separate the products of the Co_3O_4/PMS/2,6-M-PhOH system, the reaction volume was increased by 100 times to 2 L of reaction solution containing 2.88 g of PMS, 2 g of Co_3O_4, and 0.576 g of 2,6-M-PhOH. After the 1-h reaction, the catalyst was collected with a 0.22-μm filter membrane. The obtained catalyst powder was washed by ethanol to analyze the oxidative coupling products in the eluate. Then, the catalyst powder was further washed by toluene to enable analysis of the polymerization products in the eluate.

LC-MS Measurement

The products dissolved in ethanol were analyzed using liquid chromatography-mass spectrometry (LC-MS, Waters XEVO G2-XS, QTOF) equipped with a UPLC system and

an electron spray ionization source. A Waters C18 column (4.6 × 50 mm, 1.7 μm particle size) was used for UPLC separation. A mixture of methanol (containing 0.1% formic acid) and H_2O was used as the mobile phase, and the flow rate was set at 0.4 mL min^{-1}. A gradient elution method was used in the separation procedure, which increased linearly from 10:90 (methanol:H_2O) to 90:10 during 0.5 min to 4.5 min, kept at 90:10 for 1 min, and then returned to 10:90 in the last 0.5 min and maintained for 1 min to enable re-equilibration. An ESI source in the negative ionization mode was used for MS analysis. The mass calibration range was between 50 and 1200 Da and the resolution was always kept above 30000.

GPC Measurement

The products dissolved in toluene were dried at 110 ºC for 24 h in an oven to remove the solvent, producing a brown soft solid. Part of the soft solid was dissolved in tetrahydrofuran (THF), and the molecular weights and polymer dispersity index (PDI) were measured using a Waters 150C gel permeation chromatograph (GPC) equipped with microstyragel columns and an RI 2414 detector at 35 ºC. THF with a flow rate of 1.0 mL/min was used as the eluent. The molecular weights were calibrated against monodispersed polystyrene standards.

MALDI-TOF Measurement

The polymeric unit of the soft solid dissolved in THF also was analyzed using matrix-assisted laser desorption/ionization time-of-flight mass spectrometry (MALDI-TOF MS) (Atouflex Speed, Bruker Inc., USA).

C-NMR Characterization

Another part of the collected soft solid was dissolved in a mixed solvent of $CDCl_3$ and DMSO-d_6 in order to analyze its structure using nuclear magnetic resonance (NMR)

spectroscopy in a Bruker NMR spectrometer (AVANCE III 500 MHz / 54 mm). Before the C-NMR spectroscopic analysis, solvent peaks were deducted from the raw data since residual ethanol, toluene, $CDCl_3$, and DMSO-d_6 in the soft solid were inevitable. The obtained peaks of the solvents, including ethanol, toluene, $CDCl_3$, and DMSO-d_6, corresponded exactly to those in the experimental database of SDBS in AIST[2].

Galvanic cell experiments (PMS/PhOH) and analogy experiments (PhOH and KI)

To verify the direct redox reaction of PMS and PhOH on the Co_3O_4 surface, galvanic cell experiments with two control systems (the two-chamber system for the separation of PMS and PhOH, and the single-chamber system for direct contact of PMS and PhOH) were conducted. In addition, analogy experiments between PMS/PhOH and PMS/KI were conducted.

For both systems, the catalyst was loaded onto a carbon paper electrode. 100 mg of Co_3O_4 powder was first added into 10 mL of a mixture of H_2O and isopropanol in a volumetric ratio of 4:1. Then, 150 μL of Nafion solution was added to the suspension as a catalyst binder. After ultrasonic dispersion for 2 h, a homogeneous catalyst ink was obtained. The catalyst ink of 400 μL was overlaid uniformly on one side of the carbon paper sheet (2 cm × 2 cm) and then dried and stabilized at 60 ºC for 2 h in a vacuum oven. This procedure was used to fabricate uniform catalyst-coated electrodes.

In addition to the electrodes, a salt bridge of KCl was prepared and used for the two-chamber galvanic cell system. Initially, 8.94 g of KCl and 0.6 g of agar powder were added to 40 mL of DI water. After being stirred and boiled until the solution became clear, the mixture was perfused into a U-shaped glass tube of 5 mm internal diameter, and then was cooled to room temperature[3].

In both systems, the catalyst-coated electrodes were placed and fixed in the beakers. In the single-chamber (contact-type) system, the solution containing 12.5 mg/L PhOH, 20

mM borate buffer (pH = 9.0), and 20 mM Na_2SO_4 (electrolyte) was added to the beaker. After the addition of PMS (PMS:PhOH = 10:1 in molar ratio) into the beaker, the PhOH and PMS molecules were able to diffuse to the surface of the catalyst-coated electrode to contact each other and react. In the two-chamber galvanic cell system, a solution containing 12.5 mg/L PhOH, 20 mM borate buffer (pH = 9.0), and 20 mM Na_2SO_4 (electrolyte) was added into one beaker, and a solution containing 20 mM borate buffer (pH = 9.0) and 20 mM Na_2SO_4 (electrolyte) was added to another beaker. The two beakers were connected by a KCl salt bridge and a copper wire. After the addition of PMS (PMS:PhOH = 10:1 in molar ratio) to the beaker without PhOH, the PMS molecules and the PhOH molecules reacted separately at the positive and negative electrodes in each beaker in the galvanic cell, since the PMS molecules could not contact the PhOH molecules.

In the analogy experiments, the 12.5 mg/L PhOH was replaced with 108 mg/L KI.

In-situ Electron Paramagnetic Resonance (EPR) Experiments

The *in-situ* EPR experiments for radical (•OH and $SO_4^{\bullet-}$) capture were conducted by adding DMPO (the probe molecules for radical detection) to the real reaction systems with pollutant participating and tested in the reaction process (i.e., time-dependent). Specifically, DMPO was added into the solution containing 0.2 g/L Co_3O_4 (or 0.2 g/L ZVI, 0.25 mM $FeSO_4$, 0.25 mM KI) and 25 mg L^{-1} PhOH, with the DMPO concentration of 100 mM. After adding PMS (or H_2O_2) to the solution, a certain amount of the reaction solution was sampled immediately and tested in an electron paramagnetic resonance (EPR) spectrometer (JES-FA200, JEOL Co., Japan) (sweep time, 30.00 s; microwave power, 2.000 mW; field modulation amplitude, 1.000 G; and time constant, 20.48 ms). The EPR signal was recorded at given time intervals.

Nucleophilic Inhibition Experiments

To investigate the reaction mechanism, acetonitrile was used as a stronger nucleophile with respect to PhOH in order to inhibit the reaction. Once acetonitrile sheltered the oxidative intermediates (phenoxonium ions) stabilized on the catalyst surface, the reaction between the intermediates and PhOH would be suppressed. In the batch nucleophilic inhibition experiments, a mixture of DI water and acetonitrile was used to replace pure water as the solvent.

KIE Experiments

Oxidative coupling and polymerization routes entail H rearrangement and H leaving steps involving solvent H_2O. Therefore, the solvent H_2O was replaced with D_2O in the batch experiments in order to probe the influence on the reaction rate due to the kinetic isotope effect (KIE).

Real Environmental Water Anti-Interference Experiments

Tap water, lake water (Chaohu Lake, Hefei City, China), and secondary effluent from a municipal wastewater treatment plant (Wangtang Municipal Wastewater Treatment Plant, Hefei City, China) were used as the real environmental water to conduct the anti-interference experiments. Before the reaction, the lake water and secondary effluent from the municipal wastewater treatment plant were filtered with a 0.22-μm hydrophilic PTFE syringe filter to remove the solid suspensions.

Quantitation of micropollutants (BPA, 4-CP, and SMZ) in ng L^{-1} ~ μg L^{-1}

The concentrations of micropollutants in aqueous solution were quantified using liquid chromatography-mass spectrometry (LC-MS, Orbitrap Exploris 240, Thermo Fisher Inc., USA) equipped with a UPLC system and a heated electron spray ionization source. A ThermoFisher C18 column (4.6 × 10 mm, 2.1 μm particle size) was used for UPLC separation. For BPA and 4-CP, a negative ionization mode was used for MS

analysis, and a mixture of water (containing 5 mM ammonium hydroxide) and acetonitrile was used as the mobile phase. For SMZ, a positive ionization mode was used for MS analysis, and a mixture of water (containing 0.1% formic acid) and acetonitrile was used as the mobile phase. For separation procedure, the flow rate was set at 0.4 mL min^{-1}, and an isocratic elution method with a mixture ratio of 50:50 (v:v) was used. For MS procedure, the mass calibration range was in the range of 50 and 500 Da and the resolution was kept at 240000.

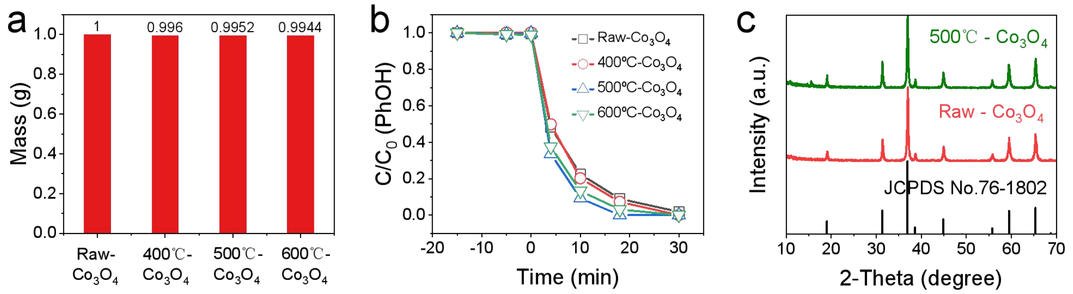

Supplementary Figure 1 | Purity verification of Co_3O_4. a, Mass loss of Co_3O_4 after annealing at different temperatures for 2 h in the air. **b,** Catalytic performance of the annealed Co_3O_4 catalysts. (PhOH = 12.5 mg/L, catalyst = 0.2 g/L, [PMS]:[PhOH] = 2 (in molar ratio), pH = 9.0, borate buffer = 20 mM). **c,** XRD patterns of the raw Co_3O_4 and the Co_3O_4 annealed at 500 ºC.

The negligible mass loss and catalytic activity change of the Co_3O_4 catalysts after annealing, as well as the correspondence of the XRD patterns to that of the standard card of Co_3O_4, confirmed that the catalysts consisted of pure Co_3O_4.

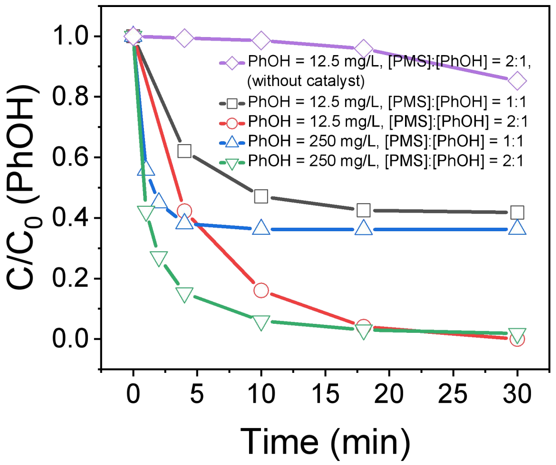

Supplementary Figure 2 | Effect of catalyst, PhOH concentrations (low and high), and dosage ratios of PMS to PhOH on PhOH removal. ([PhOH] = 12.5 mg/L (low) or 250 mg/L (high), [Co_3O_4] = 0.2 g/L (low) or 1.0 g/L (high), [PMS]:[PhOH] = 1:1 or 2:1, pH = 9, borate buffer = 0.02 M)

Catalysts play an important role in the PhOH removal process. Although the reaction rates were faster with the higher initial concentration of PhOH (250 mg/L vs 12.5 mg/L), the final removal ratio (1-C/C_0) was almost the same. At the same dosage ratio of PMS to PhOH (1:1 or 2:1), the removal ratio of PhOH was the same under different contaminant concentrations. This suggests that the removal of one PhOH consumed approximately two PMS molecules for these concentrations.

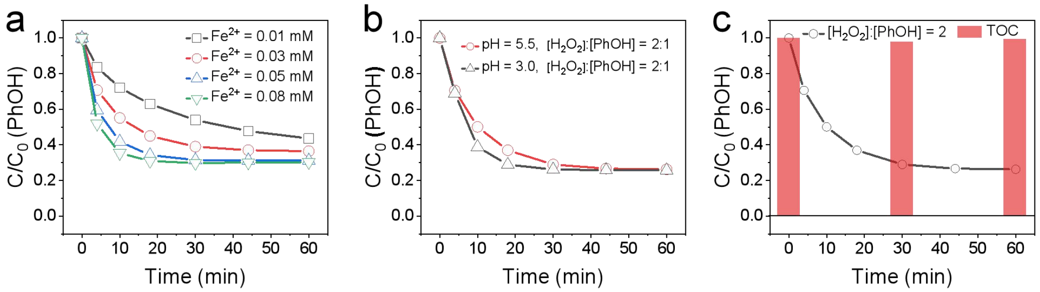

Supplementary Figure 3 | PhOH removal and TOC elimination in the Fenton system (typical representative of AOPs). a,b, Effect of Fe^{2+} concentration (**a**) and pH (**b**) on the Fenton process. **c,** TOC removal in the Fenton process with H_2O_2 to PhOH dosage ratio of 2. ([PhOH] = 25 mg/L, Fe^{2+} = 0.05 mM, [H_2O_2]:[PhOH] = 2:1 (in molar ratio), pH = 5.5)

The negligible elimination of TOC in the Fenton process indicated that mineralization in AOPs is low at an oxidant to pollutant ratio of 2:1 (low dosage of oxidizing agent used).

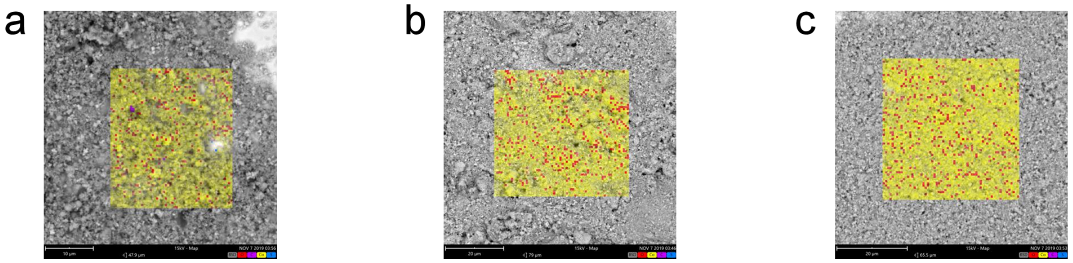

PhOH/PMS/Co_3O_4

Element Symbol	Atomic ratio (%)	Weight ratio (%)
O	38.19	24.54
Co	24.20	57.29
C	37.57	18.13
S	0.03	0.04

PhOH/Co_3O_4

Element Symbol	Atomic ratio (%)	Weight ratio (%)
O	55.44	25.75
Co	43.10	73.72
C	1.43	0.50
S	0.04	0.03

PMS/Co_3O_4

Element Symbol	Atomic ratio (%)	Weight ratio (%)
O	56.87	27.00
Co	41.38	72.36
C	1.72	0.61
S	0.03	0.03

Supplementary Figure 4 | Selected areas in SEM images and the EDS quantitative elemental analyses of Co_3O_4 reacted with PhOH and PMS (**a**), Co_3O_4 with PhOH adsorption (**b**), and Co_3O_4 with PMS adsorption (**c**). All the Co_3O_4 samples were washed with DI water and then dried before the SEM measurements.

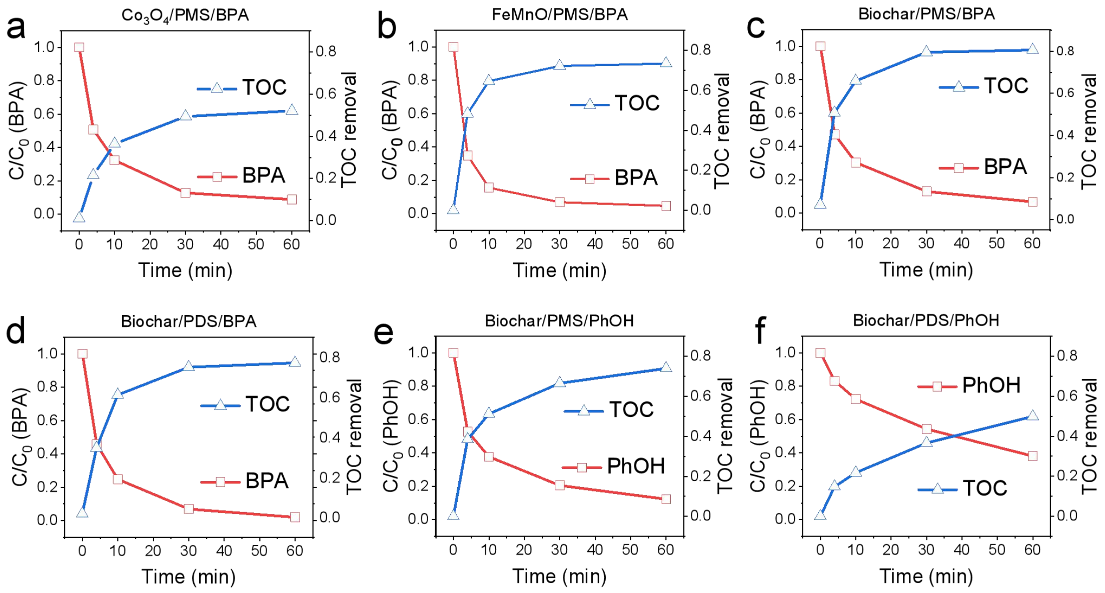

Supplementary Figure 5 | Synchronous removal process of pollutants and TOC in various persulfate-based heterogeneous catalytic oxidation systems.

a, Co_3O_4/PMS/BPA. [BPA] = 30 mg/L, [Co_3O_4] = 0.2 g/L, [PMS]:[BPA] = 2 (molar ratio), weakly acidic pH (without buffer).

b, FeMnO/PMS/BPA. [BPA] = 30 mg/L, [FeMnO] = 0.2 g/L, [PMS]:[BPA] = 2 (molar ratio), weakly acidic pH (without buffer).

c, Biochar/PMS/BPA. [BPA] = 30 mg/L, [Biochar] = 0.2 g/L, [PMS]:[BPA] = 2 (molar ratio), weakly acidic pH (without buffer).

d, Biochar/PDS/BPA. [BPA] = 30 mg/L, [Biochar] = 0.2 g/L, [PDS]:[BPA] = 2 (molar ratio), weakly acidic pH (without buffer).

e, Biochar/PMS/PhOH. [PhOH] = 25 mg/L, [Biochar] = 0.2 g/L, [PMS]:[PhOH] = 2 (molar ratio), weakly acidic pH (without buffer).

f, Biochar/PDS/PhOH. [PhOH] = 25 mg/L, [Biochar] = 0.2 g/L, [PDS]:[PhOH] = 2 (molar ratio), weakly acidic pH (without buffer).

The weakly acidic pH environment was caused by the addition of PMS or PDS, not by the pollutant suspensions (including both the pollutants and catalysts) themselves.

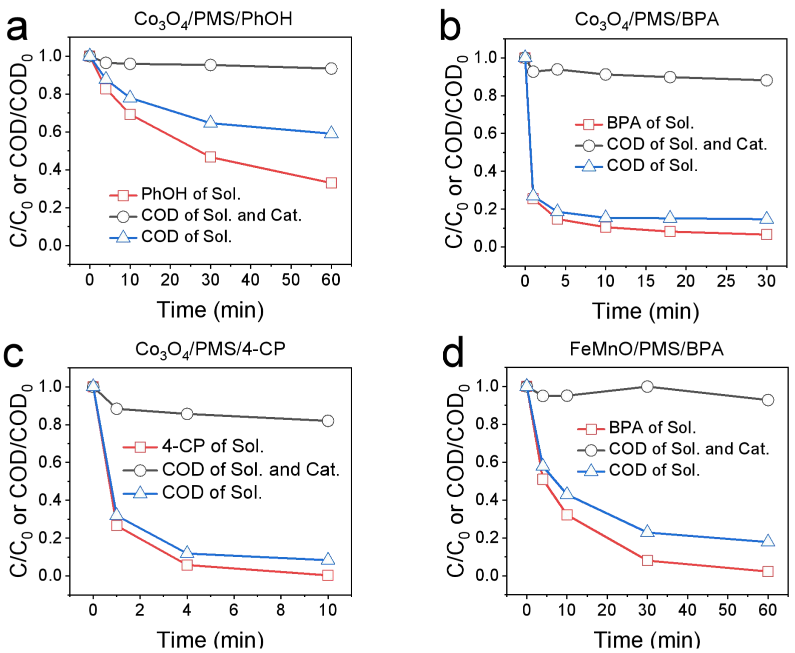

Supplementary Figure 6 | Synchronous removal process of pollutants and COD in various persulfate-based heterogeneous catalytic oxidation systems.

a, Co_3O_4/PMS/PhOH. [PhOH] = 250 mg/L, [Co_3O_4] = 1.0 g/L, [PMS]:[PhOH] = 2 (molar ratio), weakly acidic pH (without buffer).

b, Co_3O_4/PMS/BPA (pH = 9). [BPA] = 240 mg/L, [Co_3O_4] = 1.0 g/L, [PMS]:[BPA] = 2 (molar ratio), pH = 9.0, borate buffer = 20 mM.

c, Co_3O_4/PMS/4-CP (pH = 9). [4-CP] = 340 mg/L, [Co_3O_4] = 1.0 g/L, [PMS]:[4-CP] = 2 (molar ratio), pH = 9.0, borate buffer = 20 mM.

d, FeMnO/PMS/BPA. [BPA] = 240 mg/L, [FeMnO] = 1.0 g/L, [PMS]:[BPA] = 2 (molar ratio), weakly acidic pH (without buffer).

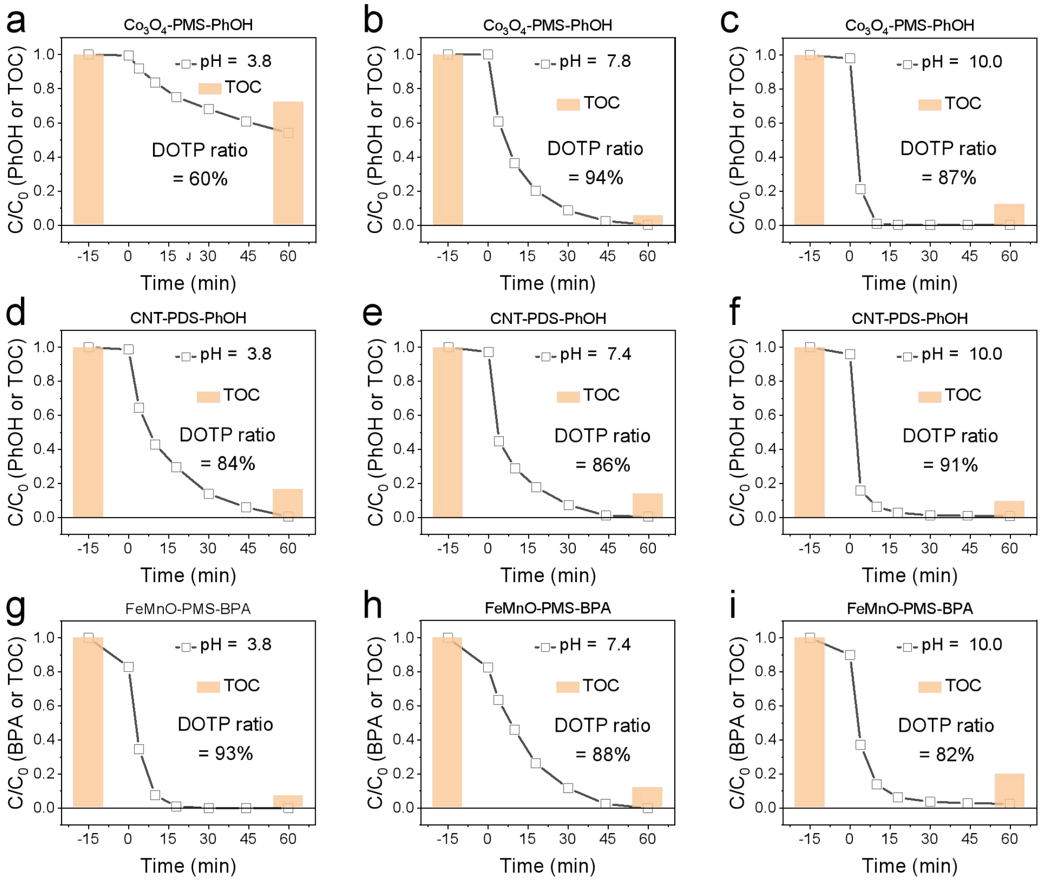

Supplementary Figure 7 | Removal efficiencies of pollutants and TOC as well as the DOTP ratios at varied solution pH values. a, b, c, Co_3O_4/PMS/PhOH system at pH of (**a**) 3.8, (**b**) 7.8, (**c**) 10.0. **d, e, f,** FeMnO/PMS/BPA system at pH of (**d**) 3.8, (**e**) 7.4, (**f**) 10.0. **g, h ,i,** CNT/PDS/PhOH system at pH of (**g**) 3.8, (**h**) 7.4, (**i**) 10.0. ([PhOH] = 25 mg L^{-1}, [BPA] = 30 mg L^{-1}, [Co_3O_4] = [FeMnO] = [CNT] = 0.2 g L^{-1}, [PMS] or [PDS] : [PhOH] or [BPA] = 2, borate buffer of 20 mM was used to control pH 7.4, 7.8, and 10.0).

Since mineralization is negligible at such a low PMS or PDS dosage, the DOTP ratio could be calculated using the following formula: the removal efficiency of TOC/the removal efficiency of pollutant. Removal efficiency was defined as (C_0-C)/C_0. The DOTP ratio is defined as the proportion of pollutant conversion accomplished via the DOTP reaction process relative to total pollutant removal.

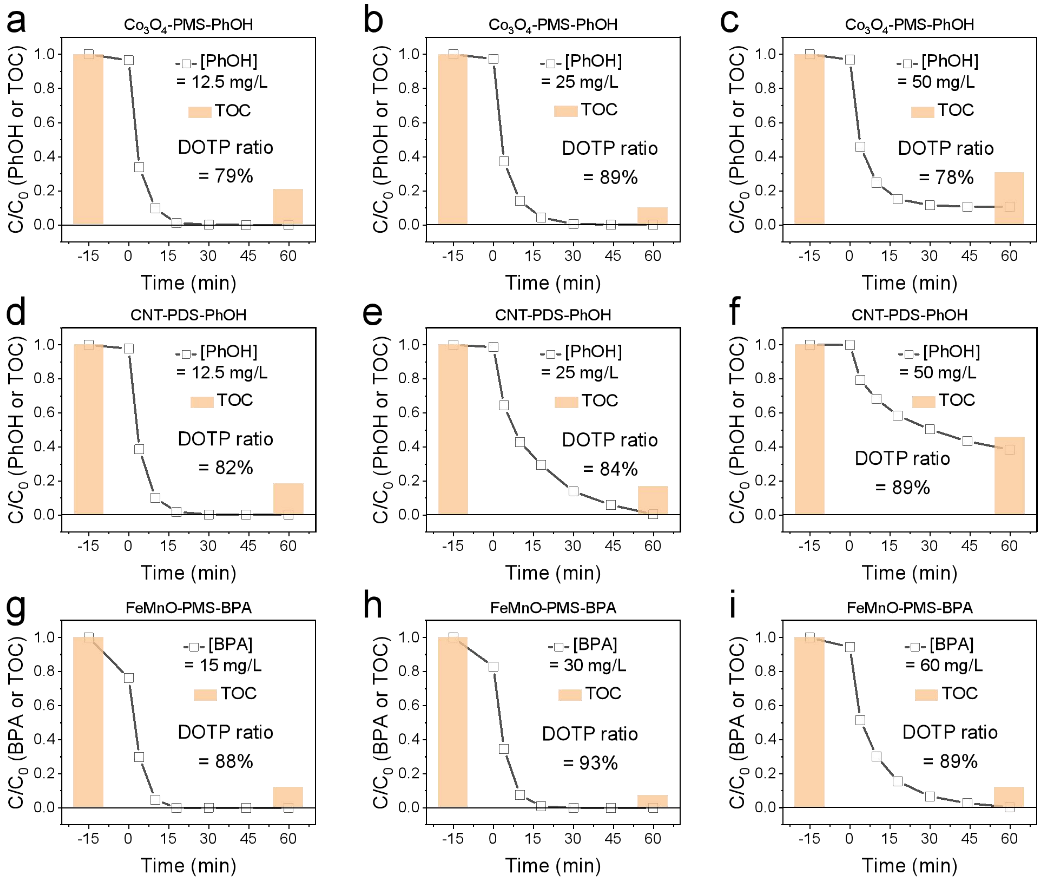

Supplementary Figure 8 | Removal efficiencies of pollutants and TOC as well as the DOTP ratios at varied pollutant concentrations. a, b, c, Co_3O_4/PMS/PhOH system at PhOH concentration of (**a**) 12.5 mg L^{-1}, (**b**) 25 mg L^{-1}, (**c**) 50 mg L^{-1}. **d, e, f,** FeMnO/PMS/BPA system at BPA concentration of (**d**) 15 mg L^{-1}, (**e**) 30 mg L^{-1}, (**f**) 60 mg L^{-1}. **g, h, i,** CNT/PDS/PhOH system at PhOH concentration of (**g**) 12.5 mg L^{-1}, (**h**) 25 mg L^{-1}, (**i**) 50 mg L^{-1} ([Co_3O_4] = [FeMnO] = [CNT] = 0.2 g L^{-1}, [PMS] or [PDS] : [PhOH] or [BPA] = 2, for Co_3O_4 system, pH = 9, borate buffer of 20 mM was used for pH control).

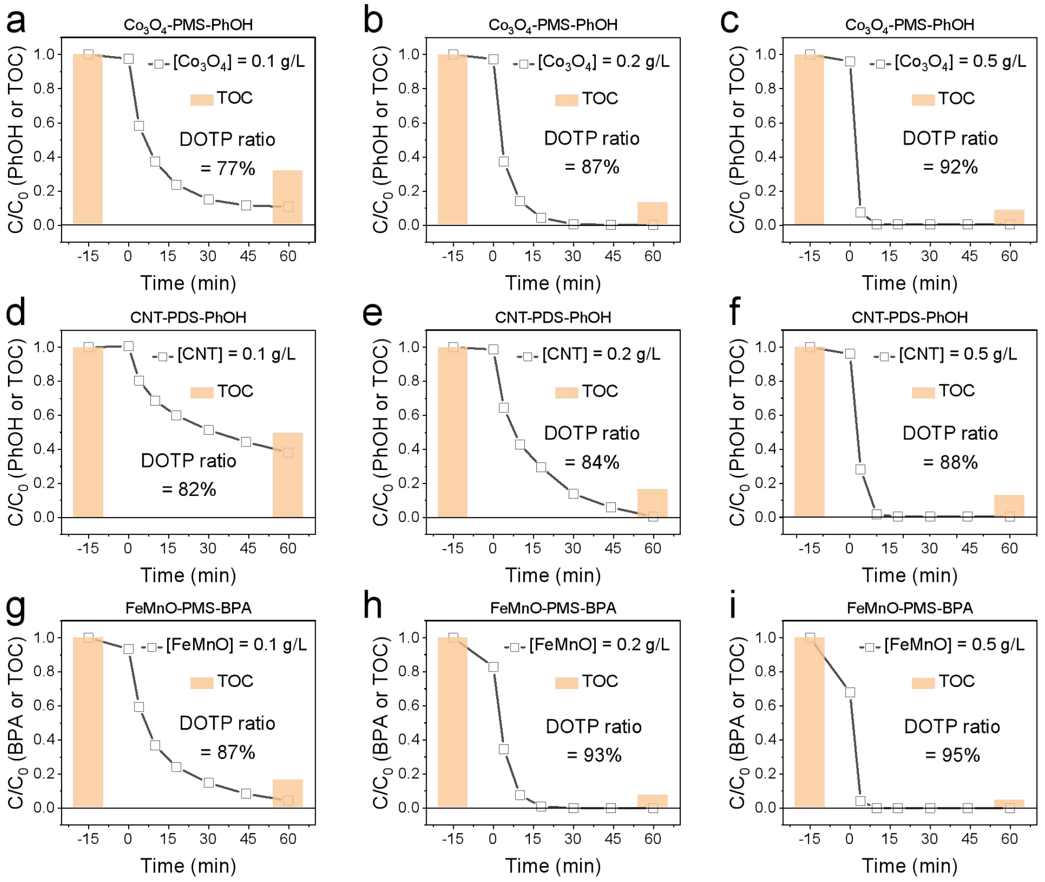

Supplementary Figure 9 | R Removal efficiencies of pollutants and TOC as well as the DOTP ratios at varied catalyst dosages. a, b, c, Co_3O_4/PMS/PhOH system at Co_3O_4 dosage of (**a**) 0.1 g L^{-1}, (**b**) 0.2 g L^{-1}, (**c**) 0.5 g L^{-1}. **d, e, f,** FeMnO/PMS/BPA system at FeMnO dosage of (**d**) 0.1 g L^{-1}, (**e**) 0.2 g L^{-1}, (**f**) 0.5 g L^{-1}. **g, h, i,** CNT/PDS/PhOH system at CNT dosage of (**g**) 0.1 g L^{-1}, (**h**) 0.2 g L^{-1}, (**i**) 0.5 g L^{-1} ([PhOH] = 25 mg L^{-1}, [BPA] = 30 mg L^{-1}, [PMS] or [PDS] : [PhOH] or [BPA] = 2, for Co_3O_4 system, pH = 9, borate buffer of 20 mM was used for pH control).

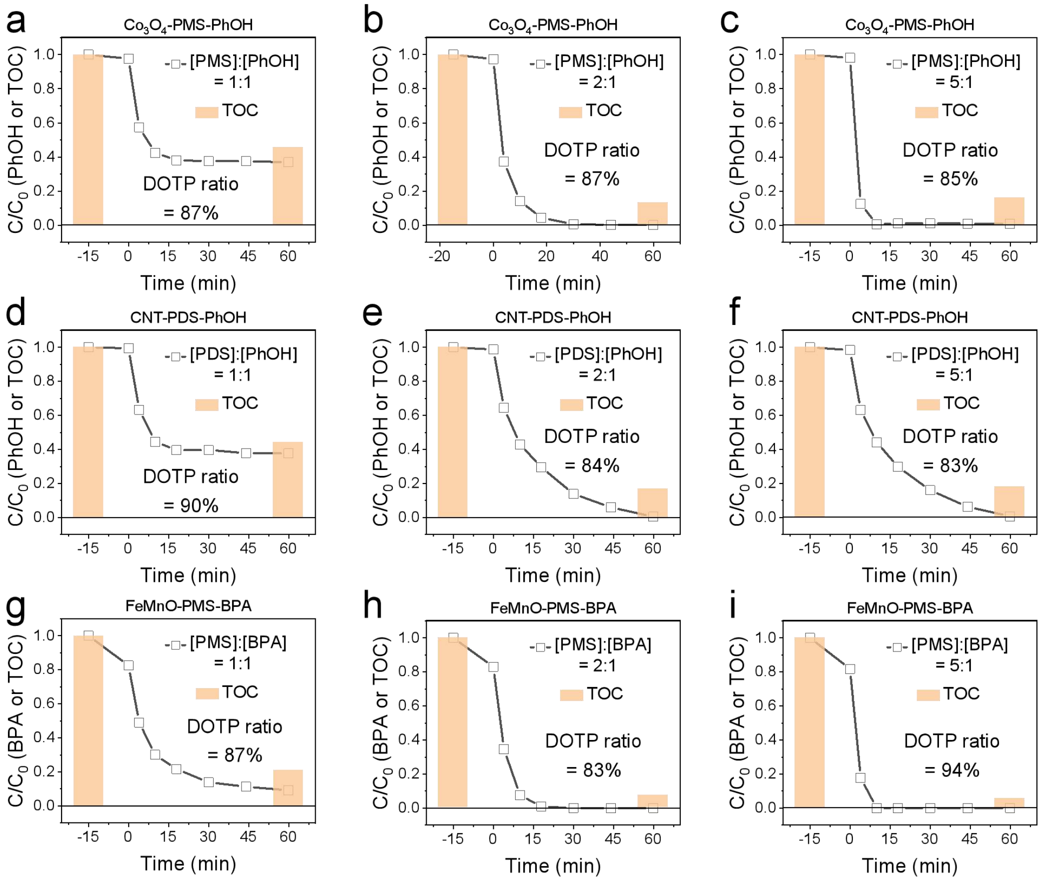

Supplementary Figure 10 | Removal efficiencies of pollutants and TOC as well as the DOTP ratios at varied oxidant concentrations. a, b, c, Co_3O_4/PMS/PhOH system at [PMS]:[PhOH] of (**a**) 1, (**b**) 2, (**c**) 5. **d, e, f,** FeMnO/PMS/BPA system at [PMS]:[BPA] of (**a**) 1, (**b**) 2, (**c**) 5. **g, h, i,** CNT/PDS/PhOH system at [PDS]:[PhOH] of (**a**) 1, (**b**) 2, (**c**) 5 ([PhOH] = 25 mg L^{-1}, [BPA] = 30 mg L^{-1}, [Co_3O_4] = [FeMnO] = [CNT] = 0.2 g L^{-1}, for Co_3O_4 system, pH = 9, borate buffer of 20 mM was used for pH control).

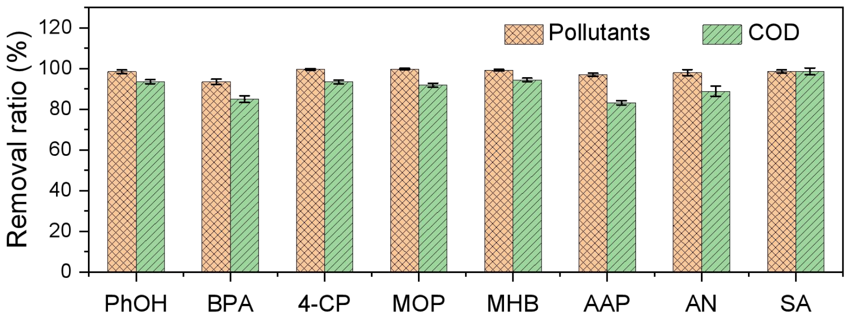

Supplementary Figure 11 | Removal ratio of the pollutants and COD in the bulk solution after a 1-h reaction in the PMS/Co_3O_4 and PDS/CNT systems containing different pollutants. [PhOH] = 250 mg/L, [BPA] = 240 mg/L, [4-CP] = 340 mg/L, [MOP] = 240 mg/L, [MHB] = 300 mg/L, [AAP] = 300 mg/L, [AN] = 242 mg/L, [SA] = 280 mg/L; Catalysts: for AAP, [Co_3O_4] = 3.0 g/L; for SA, [CNT] = 2.0 g/L; for the others, [Co_3O_4] = 1.0 g/L was used; [PMS]:[pollutant] = [PDS]:[pollutant] = 2 (in molar ratio). The PhOH concentration and the COD of the reaction solution were recorded after a 1-h reaction. BPA, bisphenol A; 4-CP, 4-chlorophenol; MOP, guaiacol; MHB, methylparaben; AAP, acetaminophen; AN, aniline; SA, sulfanilamide.

Since mineralization is negligible at such a low PMS or PDS dosage, the DOTP ratio could be calculated using the following formula: the removal efficiency of COD/the removal efficiency of pollutant. Removal efficiency was defined as (C_0-C)/C_0. The DOTP ratio is defined as the proportion of pollutant conversion accomplished via the DOTP reaction process.

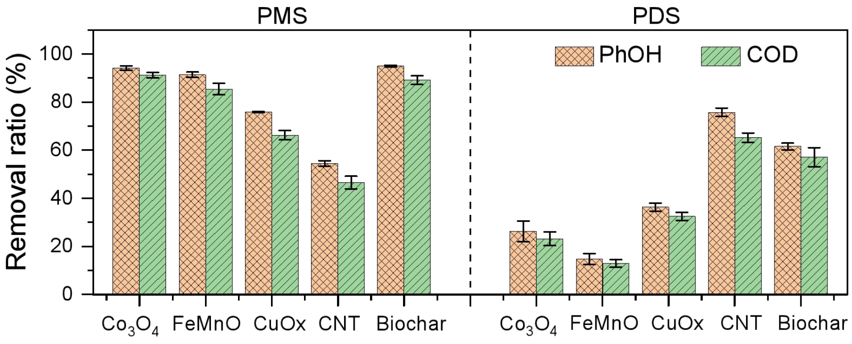

Supplementary Figure 12 | Removal ratios of PhOH and COD in the bulk solution after a 1-h reaction using different persulfates and catalysts. [PhOH] = 250 mg/L, [catalyst] = 1.0 g/L, [PMS]:[PhOH] = 2 or [PDS]:[PhOH] = 2 (in molar ratio); only for Co_3O_4, pH = 9.0, controlled with 20 mM borate buffer (the reason was described in the *Methods* section). The PhOH concentration and COD of the reaction solution were recorded after a 1-h reaction.

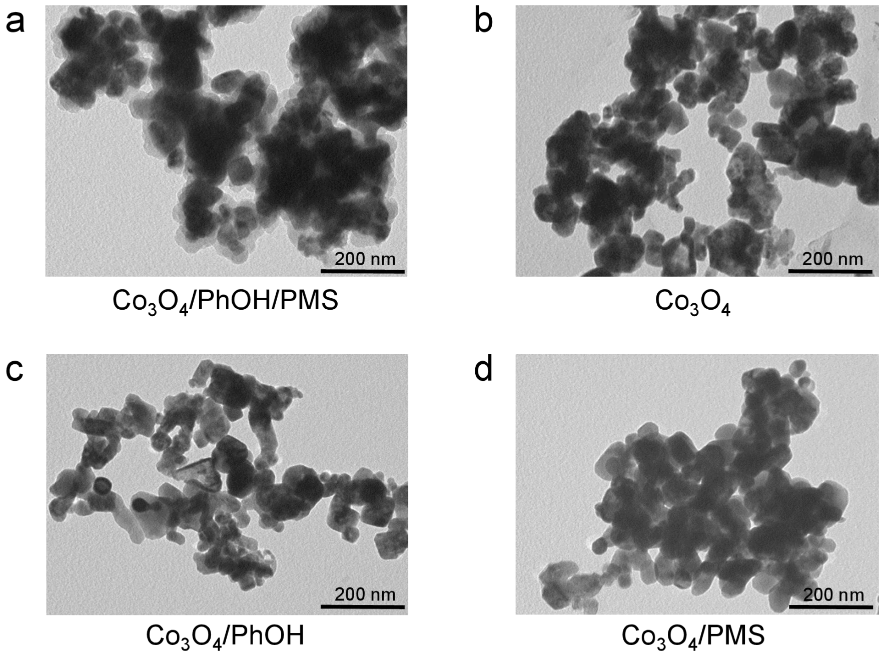

Supplementary Figure 13 | TEM images of the Co_3O_4 samples: Co_3O_4 reacted with PhOH and PMS (**a**), pristine Co_3O_4 (**b**), Co_3O_4 with PhOH adsorption (**c**), and Co_3O_4 with PMS adsorption (**d**).

Only in the reacted sample in (**a)** was there a layer of lighter contrast at the edge of the Co_3O_4 nanoparticles, which could be attributed to the organic aggregation in the DOTP reaction.

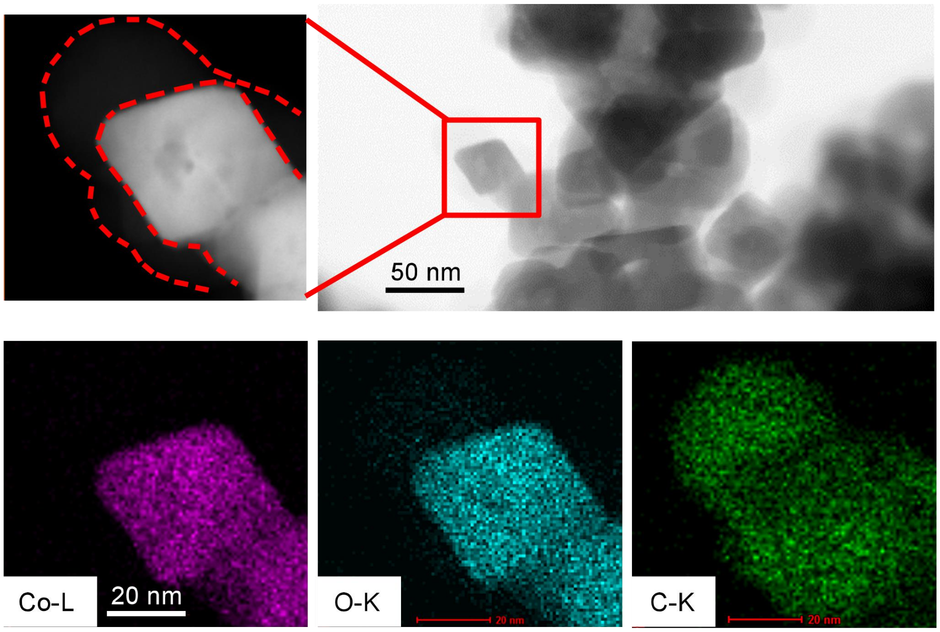

Supplementary Figure 14 | STEM-EDS elemental mapping images of the spent Co_3O_4 (Co_3O_4/PMS/PhOH).

The images show that the Co_3O_4 surface was covered by a thick layer of organic products after the reaction. The aggregated products accumulated on the catalyst surface non-uniformly, which may slow down catalyst deactivation and prolong its operation life.

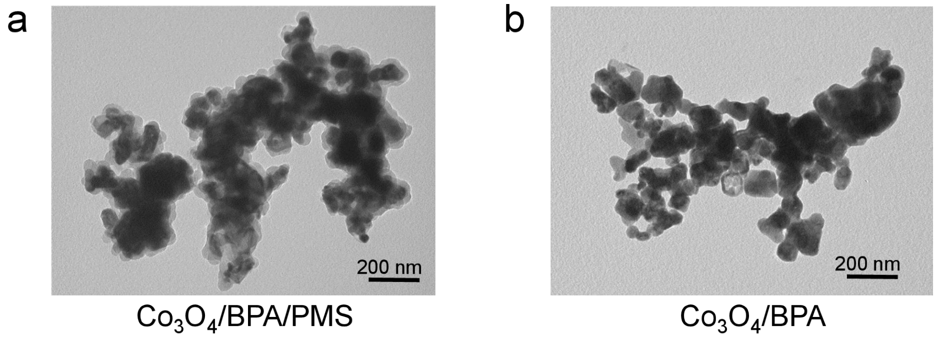

Supplementary Figure 15 | TEM images of the reacted Co_3O_4 (Co_3O_4/PMS/BPA) (**a**) and the Co_3O_4 with BPA adsorption (Co_3O_4/BPA) (**b**).

As was the case with PhOH removal with PMS, for the reaction of BPA, a layer of lighter contrast was also observed in the TEM image of the reacted Co_3O_4.

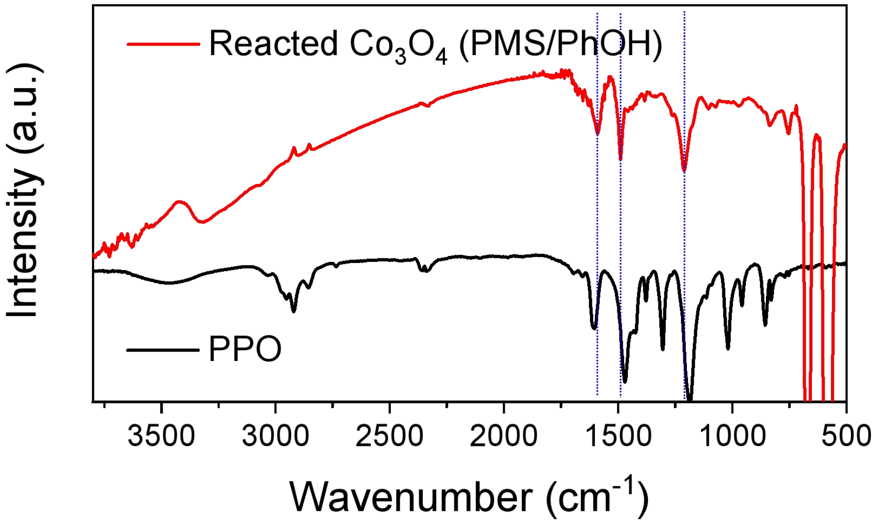

Supplementary Figure 16 | FTIR spectra of the polyphenylene oxides (PPO) and the reacted Co_3O_4 (with PhOH/PMS).

The FTIR spectra showed that the some of the peaks of the coated organics on the Co_3O_4 surface corresponded to some of the PPO peaks between 1200 to 1600 cm^{-1} (e.g., vibrational peaks of aromatic C-C and aromatic C-O-C).

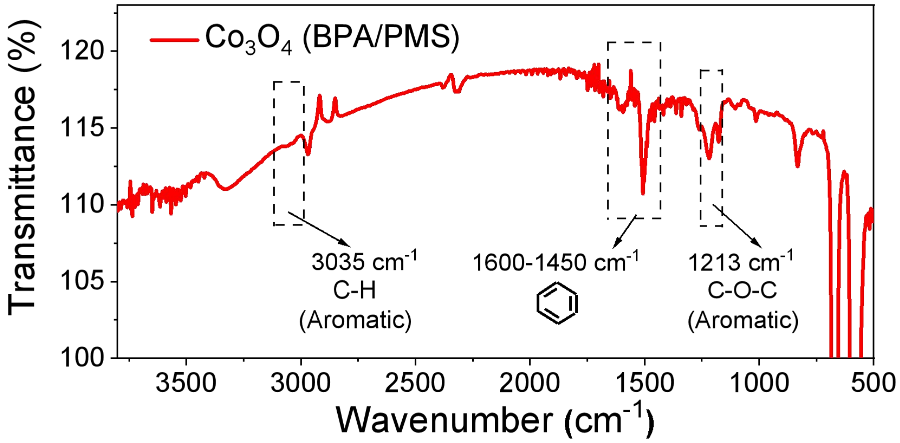

Supplementary Figure 17 | FTIR spectrum of the reacted Co_3O_4 (with BPA/PMS).

As was the case with PhOH removal with PMS, for the reaction of BPA, the vibrational peaks of aromatic C-H, aromatic C-C, and aromatic C-O-C ascribed to the characteristic signals of polyphenylene oxides were also observed on the reacted Co_3O_4[4].

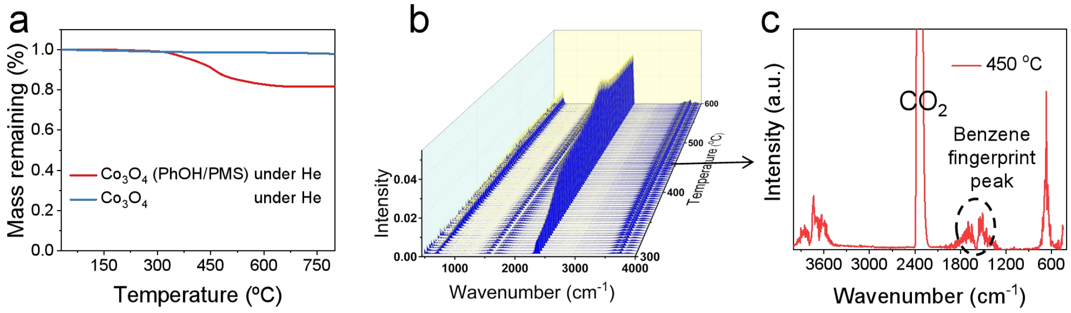

Supplementary Figure 18 | TGA-FTIR analysis of Co_3O_4 after reaction (Co_3O_4/PMS/PhOH, high-concentration group) under inert atmosphere (He). **a,** TGA curves of the pristine and spent Co_3O_4 under He. **b,** 3D-FTIR spectra of the gas products desorbed from the spent Co_3O_4 during TGA heating. **c,** Extracted 2D-FTIR spectrum at 450 ºC from **b**.

The occurrence of the major mass loss in inert atmosphere at temperatures higher than 300 ºC in the TGA curves (Fig. 2, **b** and **c** (in air) and Supplementary Figure 13) of spent Co_3O_4 indicated that the accumulated organic products were predominantly polymers. The percentage of mass lost occupied 20% of the total mass. In the reaction system, the dosage ratio in weight of PhOH to Co_3O_4 was 1:4, so the weight of PhOH was just 20% of the combined weight of PhOH and Co_3O_4. The perfectly matched results indicate that the organic pollutants in the solution were completely transferred onto the catalyst surface without any mineralization.

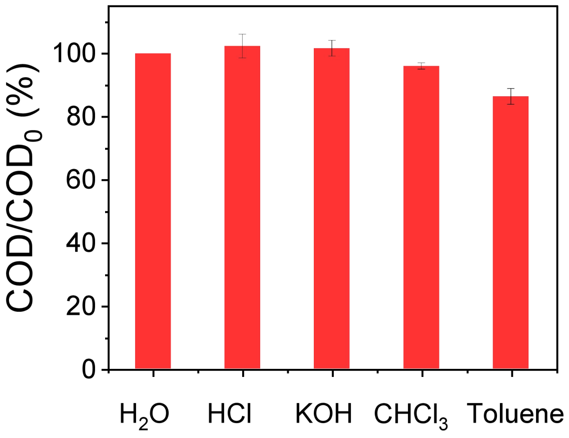

Supplementary Figure 19 | Residual COD on Co_3O_4 (Co_3O_4/PhOH/PMS) after reaction and washing with different solvents.

The small changes in COD indicated that PhOH may be transferred onto the catalyst surface mainly through a crosslinking polymerization reaction, after which the products could not be dissolved due to the crosslinked properties (the crosslinked polymer could not be dissolved in solvent for GPC, MALDI-TOF-MS, and NMR characterization).

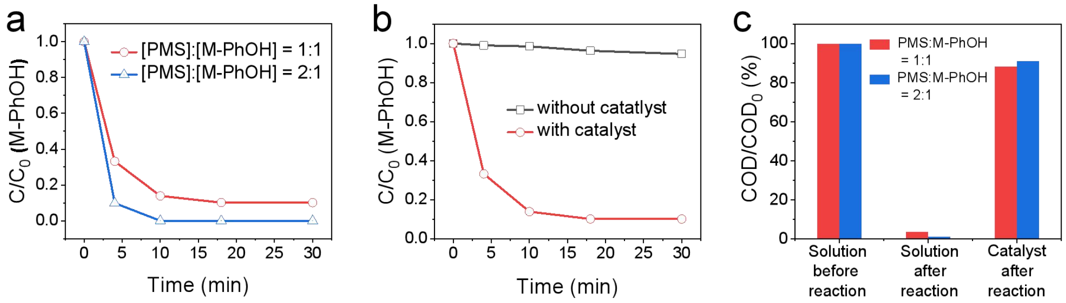

Supplementary Figure 20 | The DOTP reaction of 2,6-M-PhOH. **a,** The reaction of 2,6-M-PhOH on Co_3O_4 surface with different dosage ratios of PMS to 2,6-M-PhOH. ([2,6-M-PhOH] = 16 mg/L, [Co_3O_4] = 0.2 g/L, pH = 9.0, [PMS]:[2,6-M-PhOH] = 1:1 or 2:1, borate buffer = 20 mM). **b,** The reaction of PMS/2,6-M-PhOH with and without catalyst. ([2,6-M-PhOH] = 16 mg/L, [Co_3O_4] = 0 or 0.2 g/L, [PMS]:[2,6-M-PhOH] = 1 (in molar ratio), pH = 9.0, borate buffer = 20 mM). **c,** COD variation in the 2,6-M-PhOH reaction system. ([2,6-M-PhOH] = 288 mg/L, [Co_3O_4] = 1.0 g/L, [PMS]:[2,6-M-PhOH] = 1 or 2 (in molar ratio), pH = 9.0, borate buffer = 20 mM). M-PhOH (or 2,6-M-PhOH), 2,6-dimethylphenol.

Similar to the reaction with PhOH, in the case of 2,6-M-PhOH, the COD in the bulk solution was almost completely transferred to the catalyst surface after the reaction (Supplementary Fig. 15**c**). Supplementary Fig. 15**a** shows that at the dosage ratio of 1:1 PMS to 2,6-M-PhOH, near complete removal of 2,6-M-PhOH was achieved. Since 2,6-M-PhOH has fewer active hydrogen atoms in the benzene ring, it consumed less PMS (approximately one PMS consumed for per 2,6-M-PhOH molecule removal) in the DOTP reaction than PhOH.

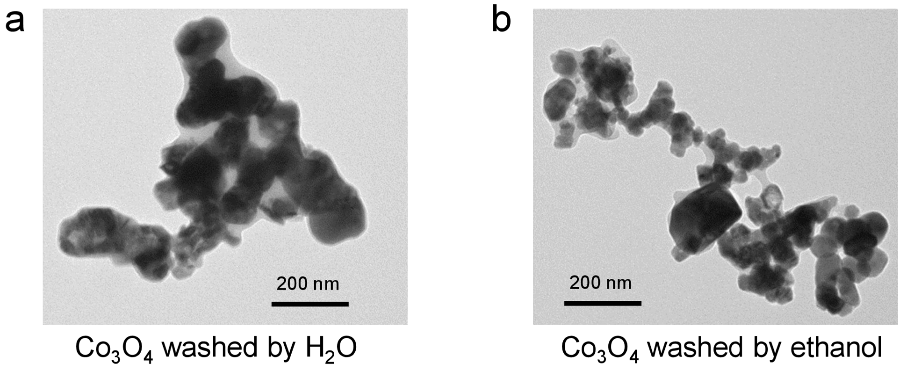

Supplementary Figure 21 | TEM images of the reacted Co_3O_4 (Co_3O_4/PMS/2,6-M-PhOH) before (**a**) and after the elution with ethanol (**b**).

In the case of 2,6-M-PhOH, a layer of lighter contrast was also observed in the TEM image of the reacted Co_3O_4, but the amount of the lighter layer (i.e., polymers) on the Co_3O_4 surface was less than that for the process using PhOH (Supplementary Fig. 9**a**). After the elution with ethanol, a residual amount of this layer remained. The polyphenylene oxides (PPO, also known as polyphenyl ethers) could not be dissolved in ethanol. Only the small molecules such as the coupling products could be dissolved in ethanol.

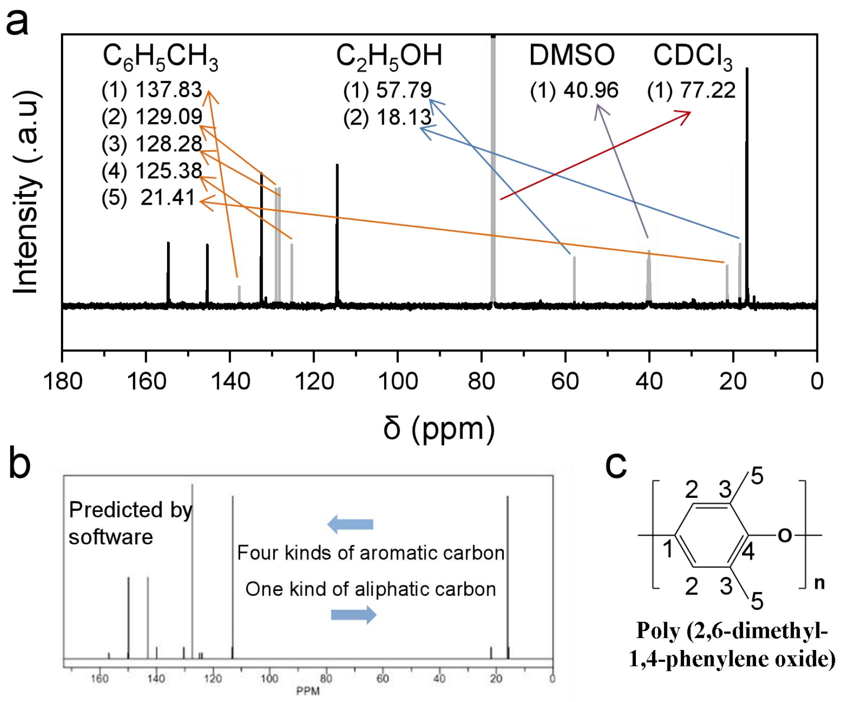

Supplementary Figure 22 | NMR-based structure analysis of the polymers produced in the Co_3O_4/PMS/2,6-M-PhOH system. **a,** Experimental C-NMR spectrum of the polymer products. **b,** Predicted C-NMR spectrum of PPO (with 15 polymeric units). **c,** The structural formula of PPO.

After deducting the solvents peaks, only five peaks remained in the experimental C-NMR spectrum, which match with PPO perfectly.

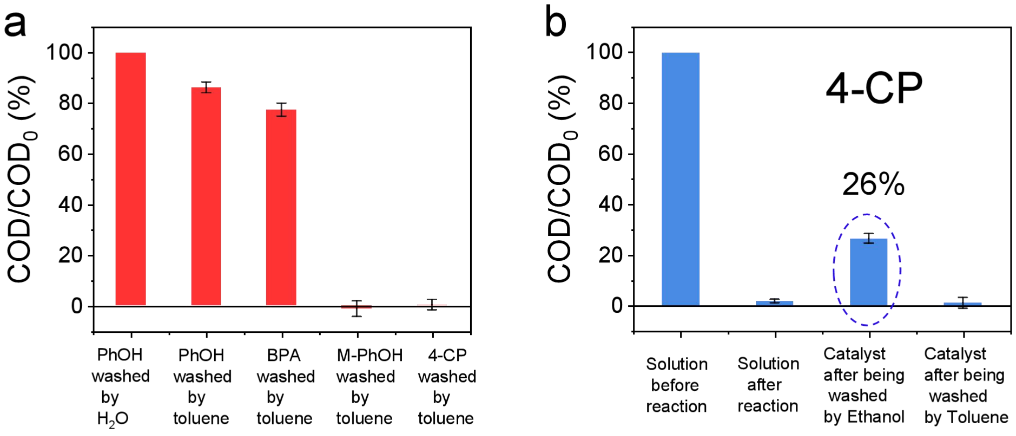

Supplementary Figure 23 | a, Residual COD of the reacted Co_3O_4 after elution with toluene for the reactions of PhOH (250 mg/L), BPA (240 mg/L), 4-CP (340 mg/L), and 2,6-M-PhOH (288 mg/L). **b,** Residual COD of the reaction solution using 4-CP (Co_3O_4/PMS/4-CP, pH = 9.0) and of the spent Co_3O_4 after elution with different solvents. 250 mg/L PhOH, 240 mg/L BPA, and 340 mg/L 4-CP had the same TOC_0 (as we defined $COD_0 = TOC_0$ earlier in the Methods section).

For PhOH and BPA, the COD was not significantly washed off the spent Co_3O_4 by toluene. This indicates that it is the polymerization pathway (86% and 77% based on COD) that mainly took place and generated the cross-linked PPO in the DOTP for PhOH and BPA, respectively. For 2,6-M-PhOH and 4-CP, the COD of polymers accumulated on the catalyst surface was 15% and 26% (Fig. 3**b** and Supplementary Fig. 12**b**) of the initial COD_0, respectively. When converted to TOC, the ratios were 17% (15% × 10/9) and 31% (26% × 13/11) (the formula was derived in the Methods section), indicating that the coupling pathway dominates in the DOTP for 2,6-M-PhOH and 4-CP.

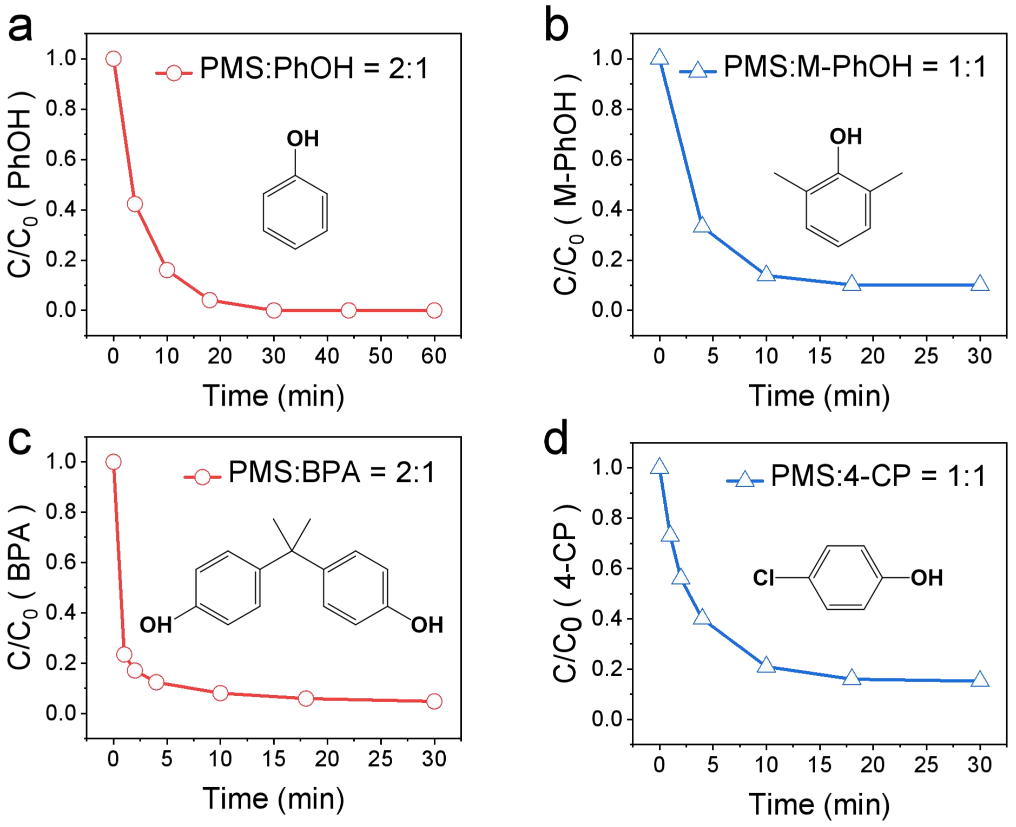

Supplementary Figure 24 | Removal processes of different organic pollutants in the PMS/Co_3O_4 system: PhOH (**a**), 2,6-M-PhOH (**b**), BPA (**c**), 4-CP (**d**). In **a** and **c**, the dosage ratio of PMS to pollutants was 2:1, while in **b** and **d**, the dosage ratio of PMS to pollutants was 1:1.

One mole of PhOH or BPA should consume approximately two moles of PMS, while one mole of 2,6-M-PhOH or 4-CP should consume one mole of PMS. There are 3 and 4 active H atoms for PhOH and BPA in their benzene rings, respectively, while for 2,6-M-PhOH and 4-CP, there are only 1 and 2 active H atoms in their benzene rings.

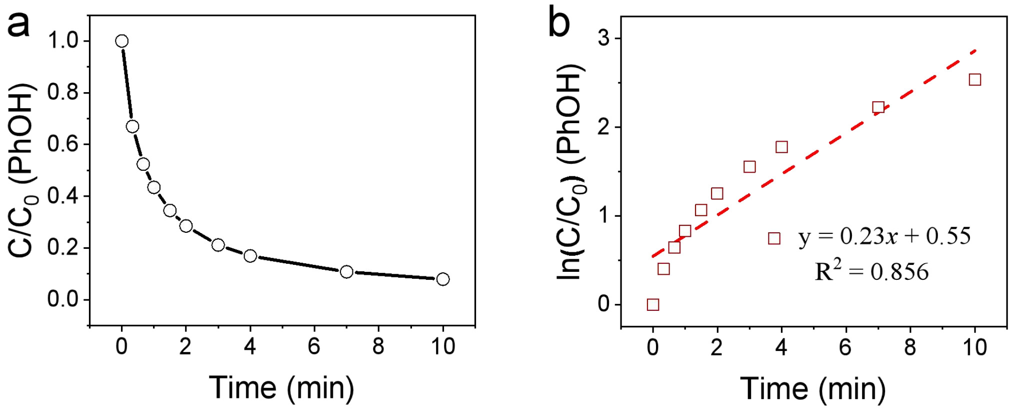

Supplementary Figure 25 | a, PhOH removal process in the DOTP reaction with the dosage ratio of PMS to PhOH set at 1.8. **b,** The corresponding pseudo-first-order kinetics of **a**. [PhOH] = 12.5 g/L, [Co_3O_4] = 0.2 g/L, [PMS]:[PhOH] = 1.8 (in molar ratio), pH = 9.0, borate buffer = 20 mM.

The low correlation coefficient (R^2 = 0.856) suggests that the pseudo-first-order kinetics is not suitable for the kinetics fitting.

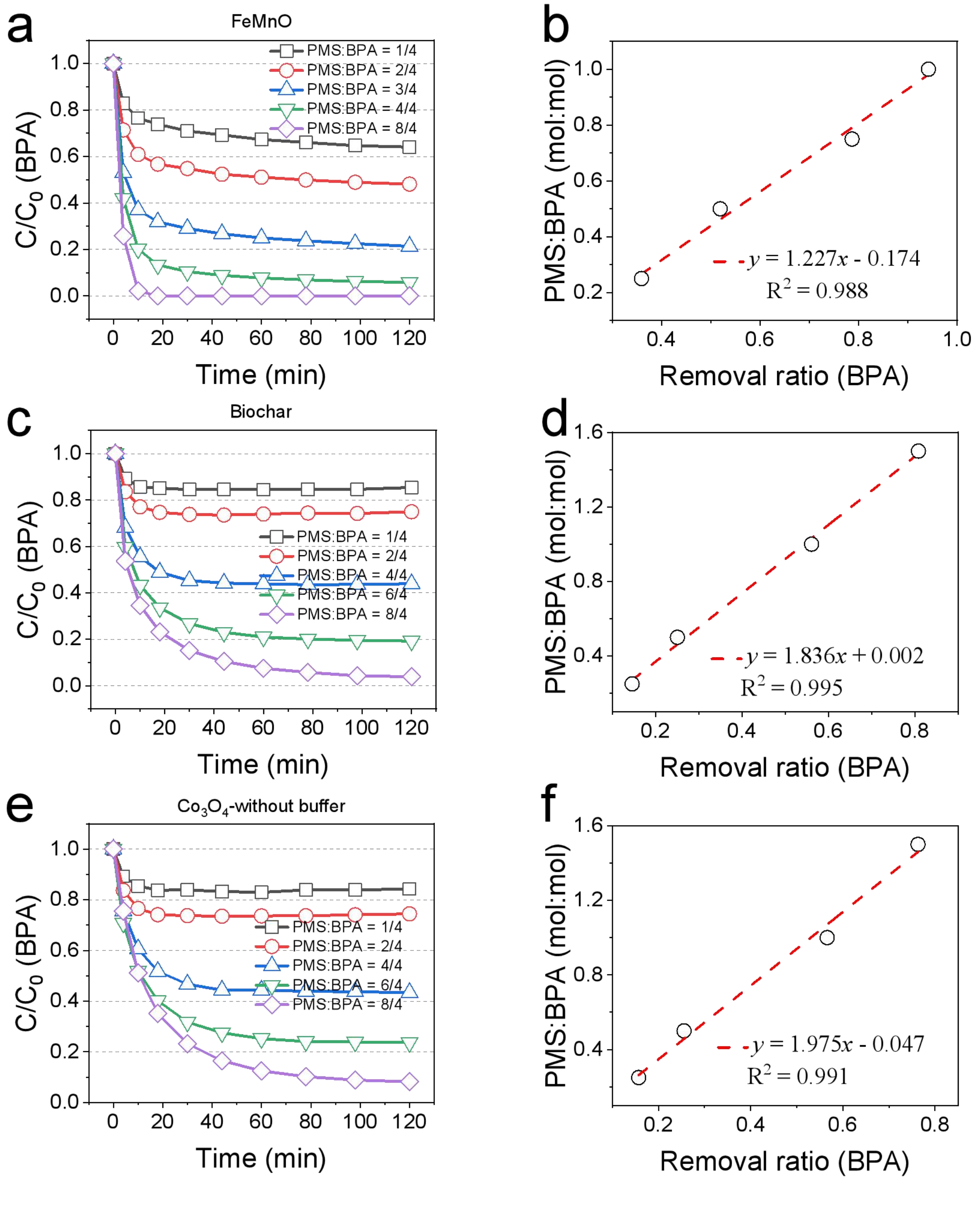

Supplementary Figure 26 | Reaction stoichiometry of PMS and BPA in the DOTP systems using different catalysts: FeMnO (**a**) and (**b**), Biochar (**c**) and (**d**), and Co_3O_4 (**e**) and (**f**) under neutral pH.

We also calculated the stoichiometric ratio of the reduced amount of PMS to the removal of BPA in these systems with different catalysts. All the obtained values were less than 2. Such low stoichiometric ratios were consistent with the redox reaction mechanism in the DOTP, suggesting a low consumption of oxidant in these processes.

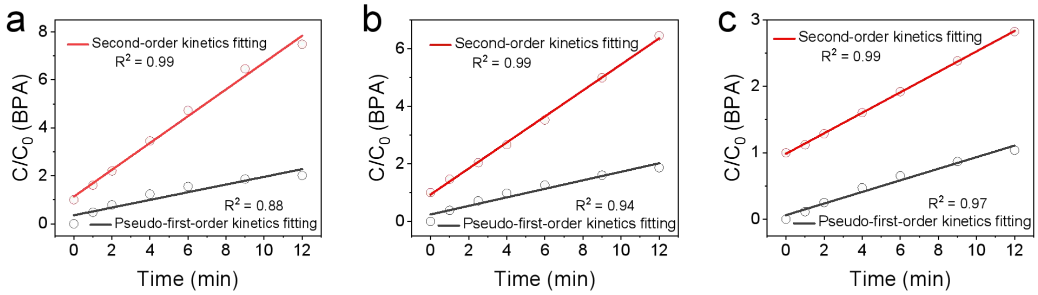

Supplementary Figure 27 | Second-order kinetics and pseudo-first-order kinetics of (**a**) FeMnO/PMS/BPA, (**b**) Biochar/PMS/BPA, (**c**) Co_3O_4/PMS/BPA.

A better fit was obtained for second-order kinetics than for pseudo-first-order kinetics in the three systems.

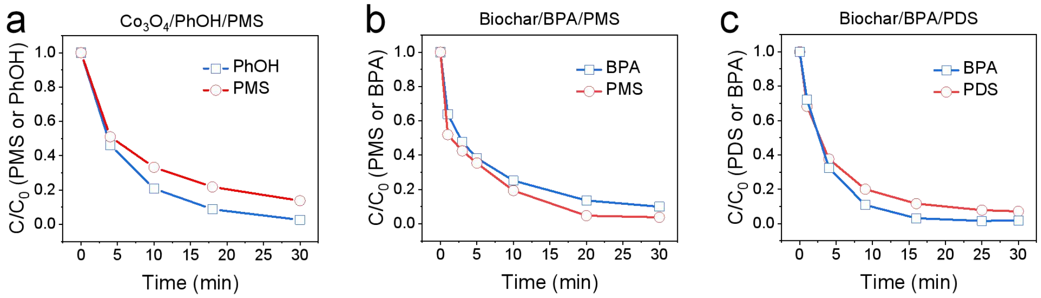

Supplementary Figure 28 | Variation of oxidant and contaminant concentrations during the reaction.

a, Co_3O_4/PhOH/PMS. [PhOH] = 12.5 mg/L, [Co_3O_4] = 0.2 g/L, [PMS]:[PhOH] = 2 (in molar ratio), pH = 9.0, borate buffer = 20 mM.

b, Biochar/BPA/PMS. [BPA] = 30 mg/L, [biochar] = 0.2 g/L, [PMS]:[BPA] = 2 (in molar ratio), near-neutral pH.

c, Biochar/BPA/PDS. [BPA] = 30 mg/L, [biochar] = 0.2 g/L, [PDS]:[BPA] = 2 (in molar ratio). near-neutral pH.

The consumption rates of oxidants and pollutants were the same once their dosage ratio was equal to the reaction stoichiometry. The simultaneous consumption of oxidants and pollutants was consistent with the second-order reaction kinetics.

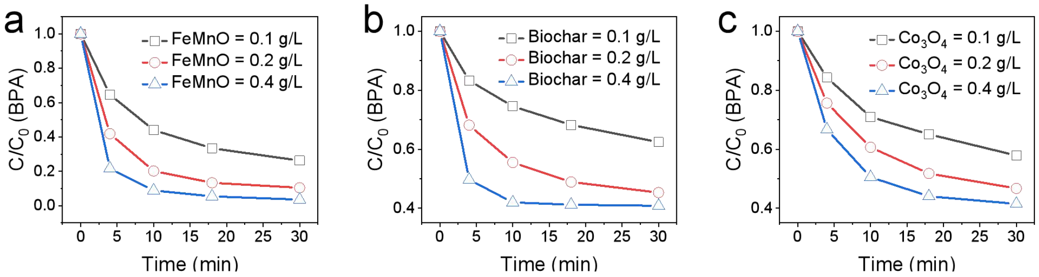

Supplementary Figure 29 | Removal process of BPA in the DOTP reaction with different catalyst concentrations: FeMnO (**a**), Biochar (**b**), Co_3O_4 (**c**). ([BPA] = 30 mg/L, [PMS]:[BPA] = 1, (in molar ratio))

The removal process reached equilibrium faster when the catalyst dose increased from 0.1 to 0.4 g/L. This result implied that the catalyst surface participates in the reaction process. It is one of the evidences that the redox reaction between PMS and BPA in DOTP depends on the catalyst surface.

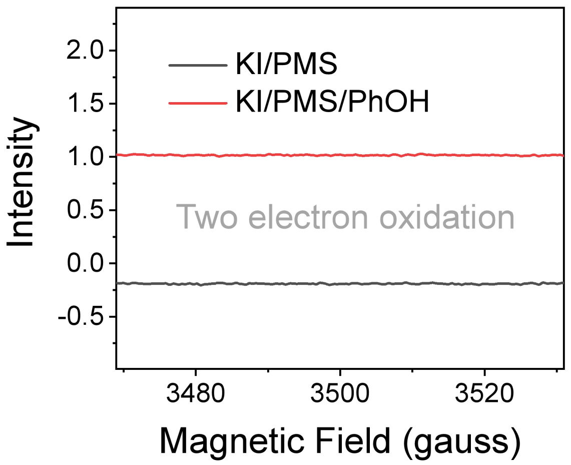

Supplementary Figure 30 | EPR spectra for $SO_4^{\bullet-}$ and •OH detection in the KI/PMS reaction process with DMPO as the radical probe molecule.

The absence of DMPO-OH or DMPO-SO_4 signal in the EPR spectra indicated no $SO_4^{\bullet-}$ and •OH formation in the KI/PMS reaction process, which corresponded to the two electron oxidation mechanism, where the PMS was reduced to SO_4^{2-} by I^- (for the reduction of PMS, there is one-electron process involving $SO_4^{\bullet-}$ and •OH formation and a two-electron process involving SO_4^{2-} formation).

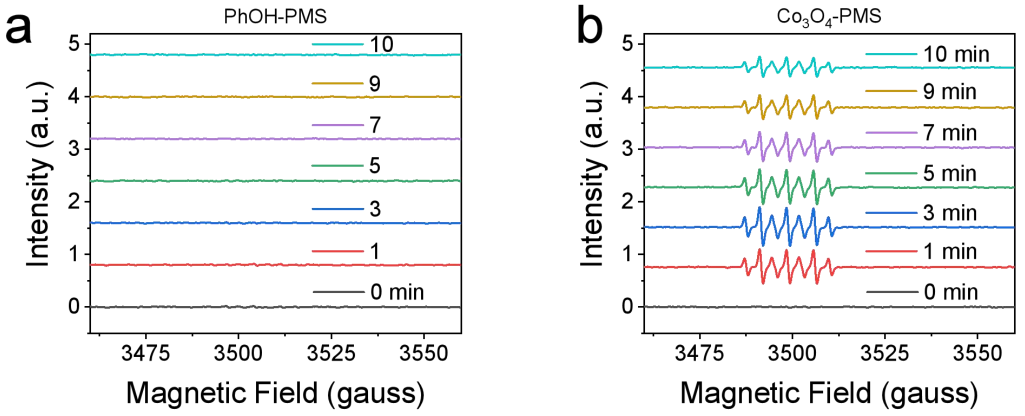

Supplementary Figure 31 | a,b, Time-dependent EPR spectra of (**a**) PhOH/PMS and (**b**) Co_3O_4/PMS systems with DMPO as the probe molecule ([PhOH] = 25 mg L^{-1}, [Co_3O_4] = 0.2 g L^{-1}, [PMS]:[PhOH] = 2, pH = 9, borate buffer = 20 mM, [DMPO] = 100 mM).

DMPO could not be directly oxidized by PMS without Co_3O_4 (Fig. S**31a**), indicating the catalytic function of the Co_3O_4 surface for the reaction between PMS and DMPO in the Co_3O_4/PMS/PhOH system. The intensity of DMPOX signals in the Co_3O_4/PMS system (Fig. S**31b**) was higher than that in the Co_3O_4/PMS/PhOH system (Fig. **4g**), indicating that the competitive reactions between PMS/PhOH and PMS/DMPO were catalyzed by the Co_3O_4 surface.

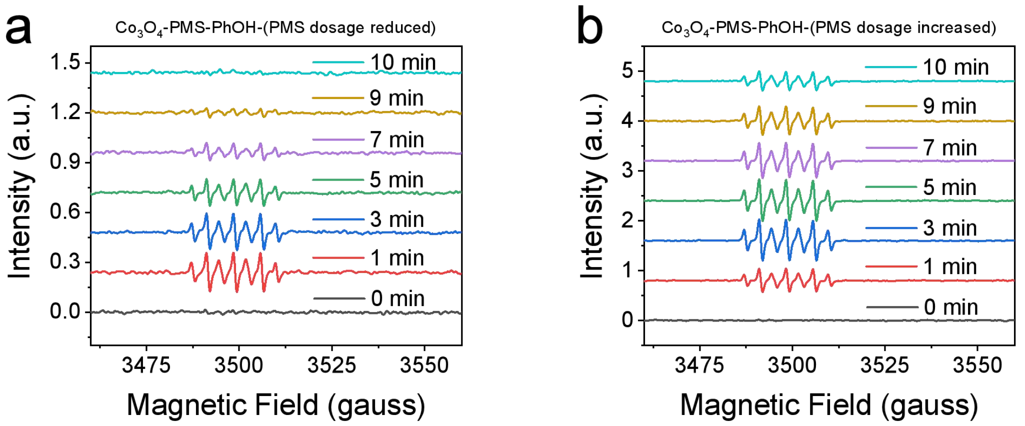

Supplementary Figure 32 | Effect of PMS concentration on the time-dependent EPR spectra of the Co_3O_4/PMS/PhOH system with DMPO as the probe molecule. a, [PMS]:[PhOH] = 1. **b,** [PMS]:[PhOH] = 5. ([PhOH] = 25 mg L^{-1}, [Co_3O_4] = 0.2 g L^{-1}, pH = 9, borate buffer = 20 mM, [DMPO] = 100 mM)

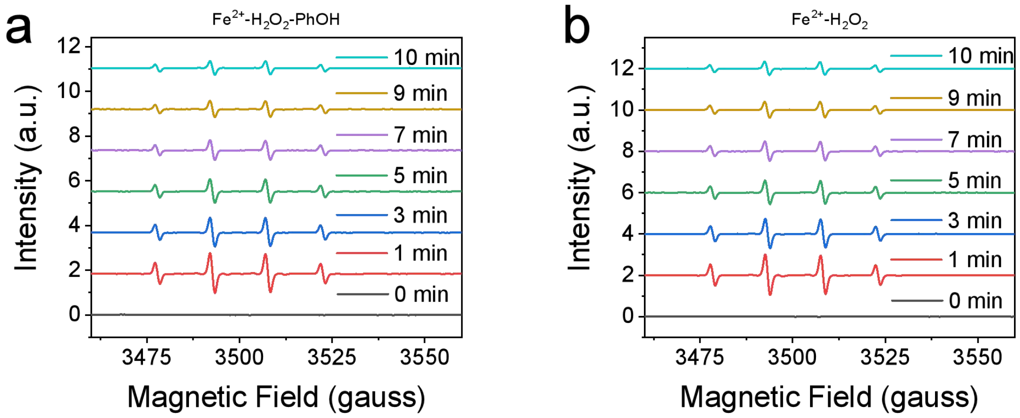

Supplementary Figure 33 | a,b, Time-dependent EPR spectra of (**a**) Fe^{2+}/H_2O_2/PhOH and (**b**) Fe^{2+}/H_2O_2 systems with DMPO as the probe molecule. ([PhOH] = 25 mg L^{-1}, [Fe^{2+}] = 0.05 mM, [H_2O_2]:[PhOH] = 2, [DMPO] = 100 mM)

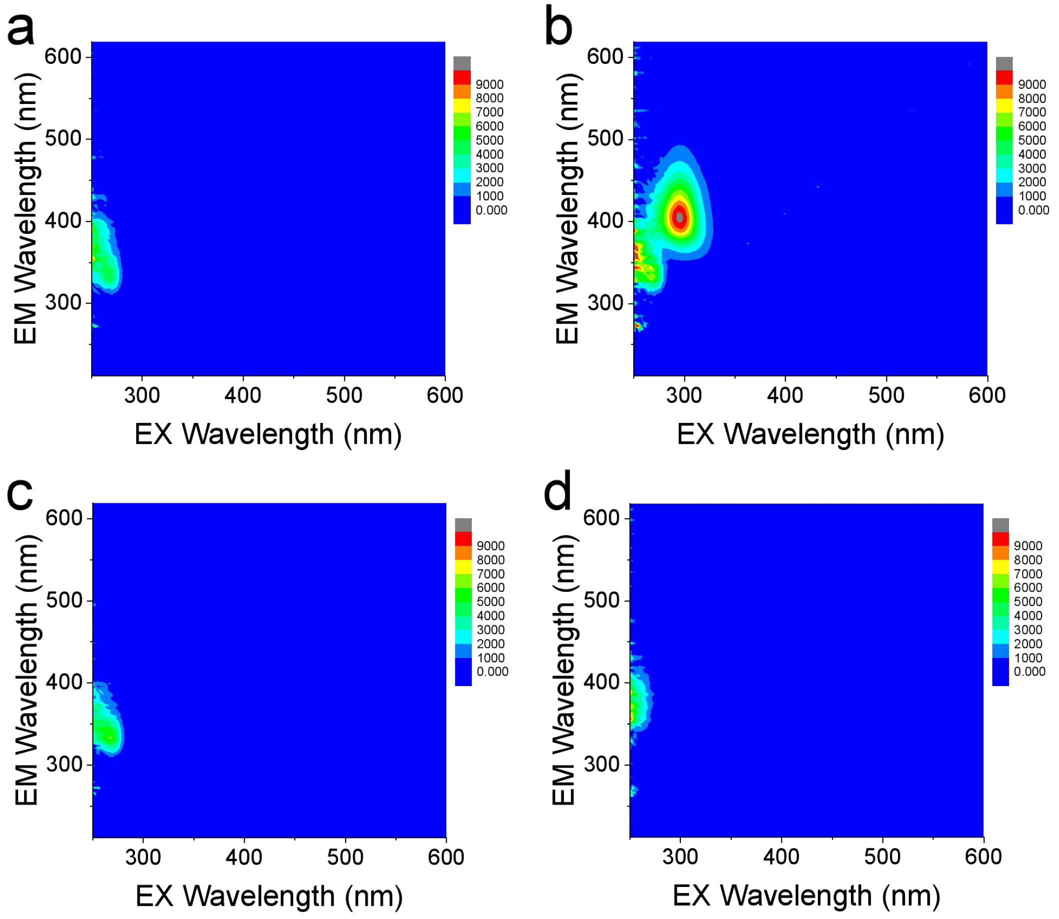

Supplementary Figure 34 | Quantitative fluorescence detection of the radicals by benzoic acid (BA) in the Fenton and Co_3O_4/PMS/PhOH systems. a,b, EEMs of Fenton system (**a**) before and (**b**) after H_2O_2 addition. **c,d,** EEMs of Co_3O_4/PMS/PhOH system (**c**) before and (**d**) after PMS addition. ([PhOH] = 12.5 mg L^{-1}, [Co_3O_4] = 0.2 g L^{-1}, [Fe^{2+}] = 0.05 mM, [PMS]:[PhOH] = [H_2O_2]:[PhOH] = 2, [BA] = 100 mg L^{-1}, for Co_3O_4 system, pH = 9.0, borate buffer = 20 mM). EEMS, excitation-emission matrix spectra.

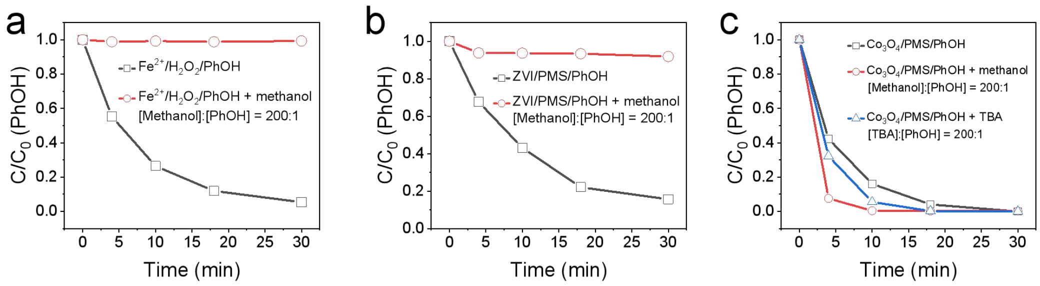

Supplementary Figure 35 | Effect of radical scavengers (methanol and tert-butyl alcohol, TBA) on the typical AOP systems (Fe^{2+}/H_2O_2 and ZVI/PMS) and the Co_3O_4/PMS/PhOH DOTP system: Fe^{2+}/H_2O_2/PhOH (**a**), ZVI/PMS/PhOH (**b**), and Co_3O_4/PMS/PhOH (**c**).

Unlike the complete inhibition of the radical-based AOPs, methanol and tert-butyl alcohol (200 times the molar ratio of PhOH) had no inhibitory effect on the Co_3O_4/PMS/PhOH reaction process, which indicated that no radicals were involved.

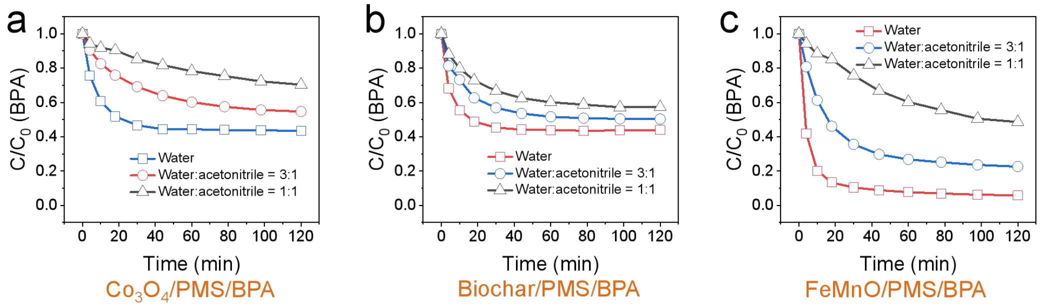

Supplementary Figure 36 | Nucleophilic inhibition experiments on different catalysts: Co_3O_4 (**a**), Biochar (**b**), FeMnO (**c**).

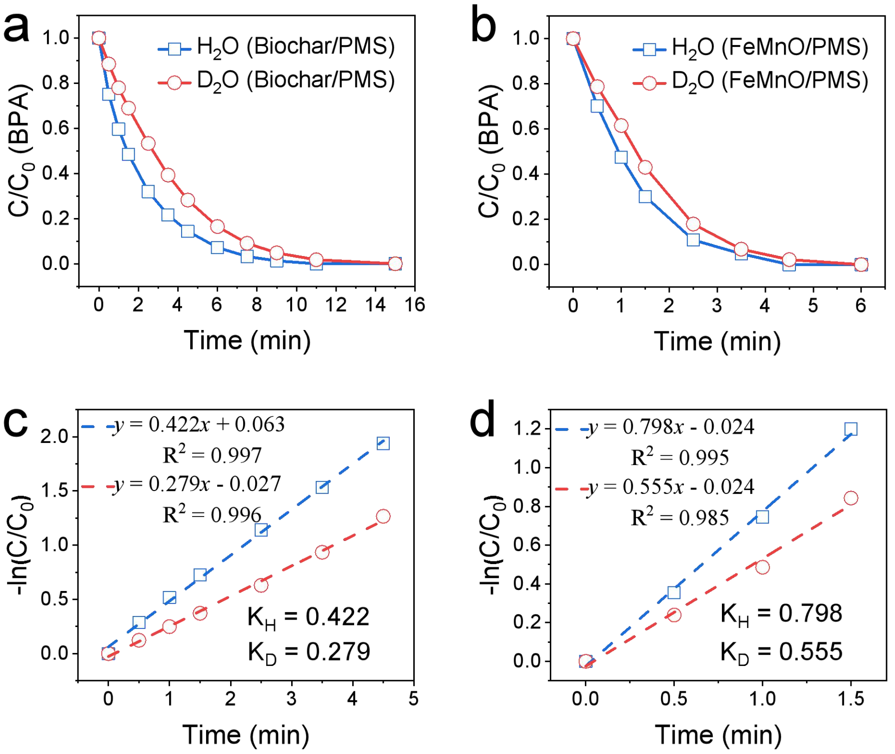

Supplementary Figure 37 | Identification of the KIE in different reaction systems. **a,c,** PMS/Biochar/BPA system. [BPA] = 30 mg/L, [biochar] = 0.2 g/L, [PMS]:[BPA] = 10 (in molar ratio). **b,d,** PMS/FeMnO/BPA system. [BPA] = 30 mg/L, [FeMnO] = 0.2 g/L, [PMS]:[BPA] = 10 (in molar ratio).

The dosage ratio of PMS to BPA employed was much larger than the reaction stoichiometry so that the PMS was in excess. Therefore, the reaction process could be fit to the pseudo-first-order kinetics model. The calculated KIE values of the biochar/PMS/BPA system and the FeMnO/PMS/BPA system were ~ 1.513 (K_H/K_D = 0.422/0.279) and ~ 1.438 (K_H/K_D = 0.798/0.555), respectively. All the KIE values in Supplementary Fig. 28 are in the range of secondary kinetic isotope effects, indicating a conversion from *sp*^{3}-C to *sp*^{2}-C in the solvent condition[5-8]. The conversion is reflected in the H rearrangement and H leaving steps in the processes of PR and CR products formation, respectively.

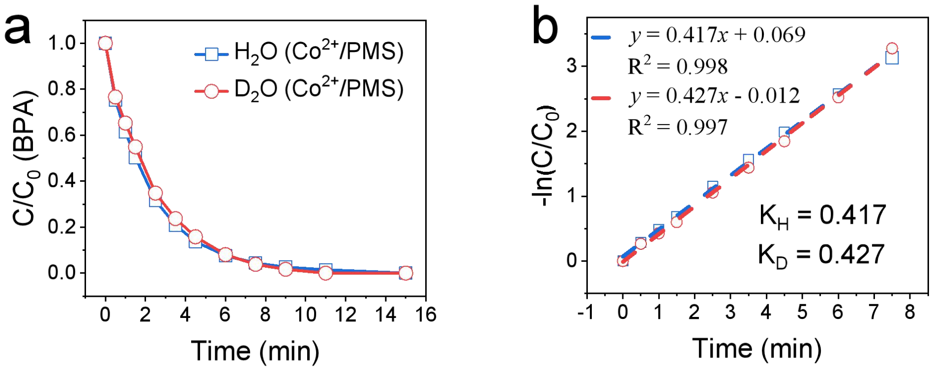

Supplementary Figure 38 | Identification of the KIE effect in the homogeneous Fenton-like process. **a,** Removal processes of BPA in H_2O and D_2O. **b,** Corresponding kinetic constants of **a**. [BPA] = 30 mg/L, [Co^{2+}] = 0.1 mM, [PMS]:[BPA] = 10 (in molar ratio).

The calculated KIE value is approximately 1 (K_H/K_D = 0.417/0.427), indicating no KIE in the homogeneous Fenton-like process. Therefore, this process is dependent on the sulfate/hydroxyl radicals and does not involve the conversion from *sp*^{3}-C to *sp*^{2}-C hybridization as in the DOTP pathways.

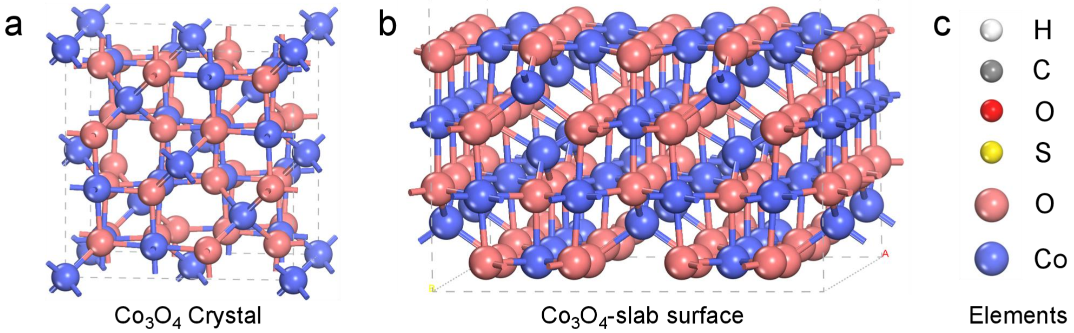

Supplementary Figure 39 | Optimized structures of the (**a**) cubic Co_3O_4 crystal and its (**b**) slab and (**c**) symbol of elements for representation.

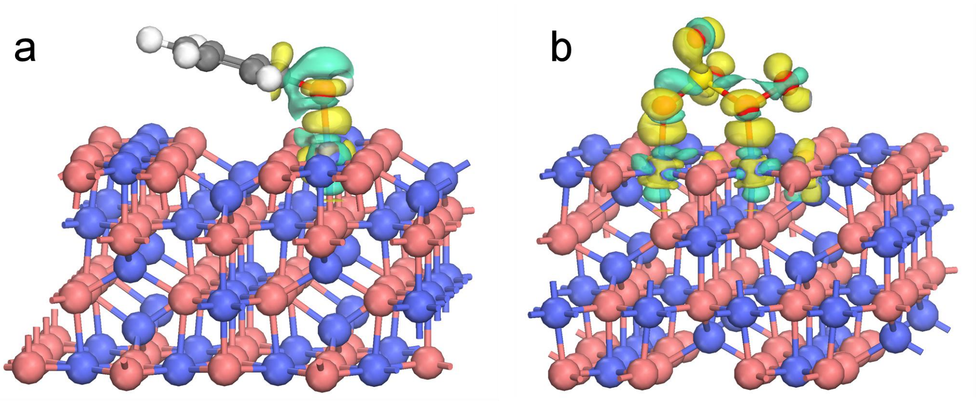

Supplementary Figure 40 | Theoretical calculations of PhOH and PMS adsorption on Co_3O_4 surface. a,b, Charge density difference of (**a**) PhOH on Co_3O_4 surface and (**b**) PMS on Co_3O_4 surface. The yellow and blue isosurfaces represent charge accumulation and depletion in the space, respectively. The isovalue is 0.02 au. Blue, Co; white, H; grey, C; red, O; yellow, S.

The charge density difference results show that both PMS and PhOH have electron cloud flow and rearrangement on the Co_3O_4 surface, indicating the activation of PMS and PhOH by the catalyst surface. Specifically, the partial electron cloud flowed from PhOH to the Co_3O_4 surface, and the partial electron cloud flowed from Co_3O_4 to PMS. This result indicates that PhOH is negatively activated and PMS is positively activated in the view of standard electrode potential to increase the redox potential difference, which is beneficial for the subsequent surface-catalyzed direct oxidation.

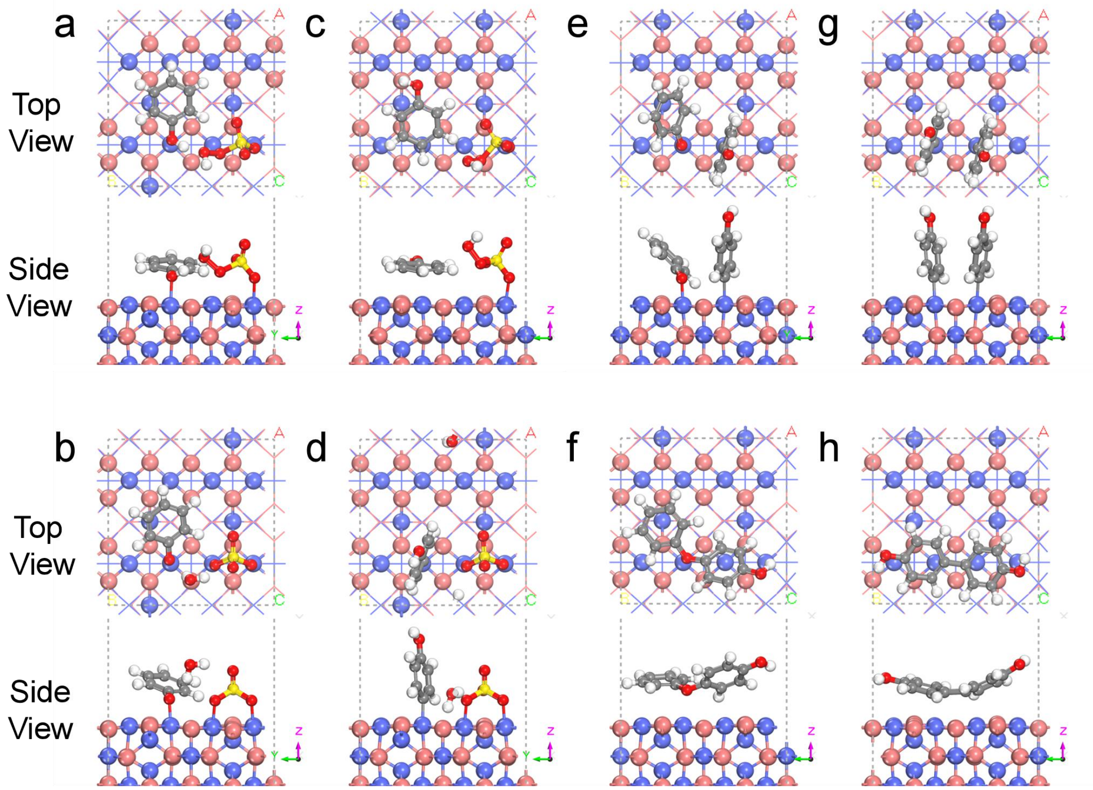

Supplementary Figure 41 | Optimized structures in the phenol reaction pathway of the DOTP (on Co_3O_4 surface).

a, Co_3O_4 + PhO-OH + PMS. **b,** Co_3O_4 + PhO-O + H_2O/SO_4.

c, Co_3O_4 + PhO-CH + PMS. **d,** Co_3O_4 + PhO-C + H_2O/SO_4.

e, Co_3O_4 + PhO-O + PhO-C. **f,** Co_3O_4 + PhO-O-C-PhO.

g, Co_3O_4 + PhO-C + PhO-C. **h,** Co_3O_4 + PhO-C-C-PhO.

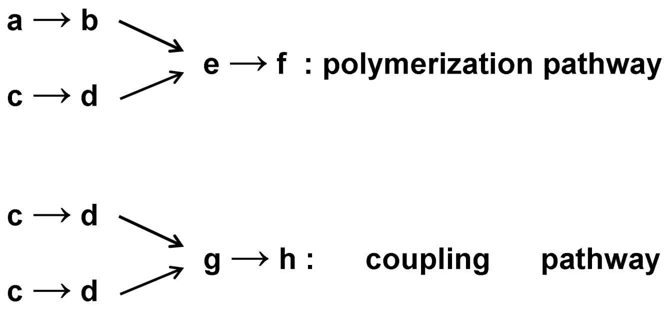

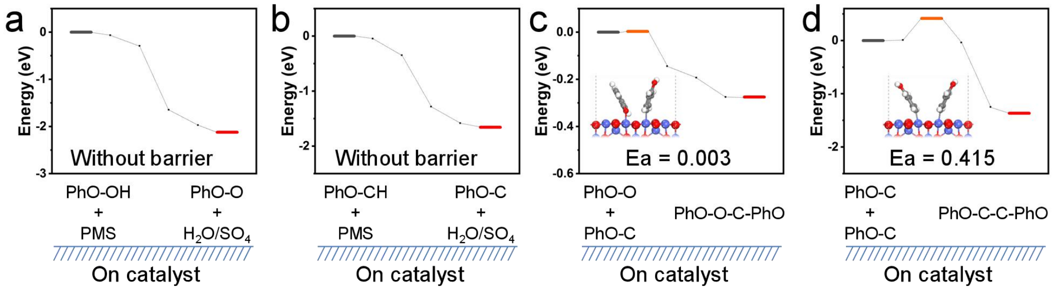

Supplementary Figure 42 | Calculated kinetics of the reaction pathway of PhOH in the DOTP on the Co_3O_4 surface.

a, Co_3O_4 + PhO-OH + PMS → Co_3O_4 + PhO-O + H_2O/SO_4.

b, Co_3O_4 + PhO-CH + PMS → Co_3O_4 + PhO-C + H_2O/SO_4.

c, Co_3O_4 + PhO-O + PhO-C → Co_3O_4 + PhO-O-C-PhO.

d, Co_3O_4 + PhO-C + PhO-C → Co_3O_4 + PhO-C-C-PhO.

There was almost no energy barrier for the oxidation of phenol and the production of phenol intermediates on the Co_3O_4 surface. This was due to the activation of PMS and PhOH and the stabilization of the oxidative phenol intermediates on the catalyst surface. In the subsequent reactions of the intermediates, the polymerization route dominated the oxidative coupling route, since the activation energy (Ea) of the former route was much smaller than that of the latter (shown in **c** and **d**).

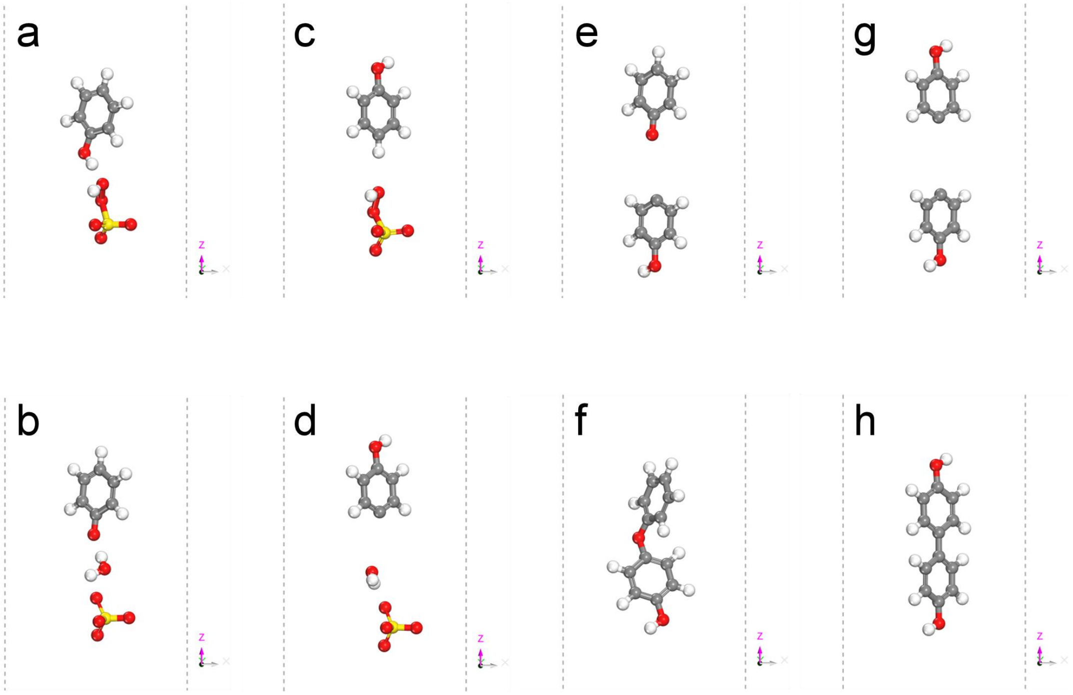

Supplementary Figure 43 | Optimized structures in the phenol reaction pathway of the control process without the Co_3O_4 surface.

a, PhO-OH + PMS. **b,** PhO-O + H_2O/SO_4.

c, PhO-CH + PMS. **d,** PhO-C + H_2O/SO_4.

e, PhO-O + PhO-C. **f,** PhO-O-C-PhO.

g, PhO-C + PhO-C. **h,** PhO-C-C-PhO.

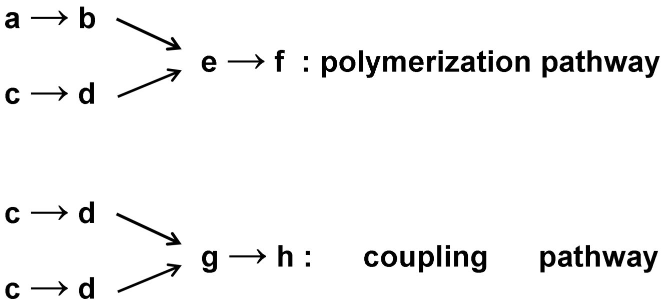

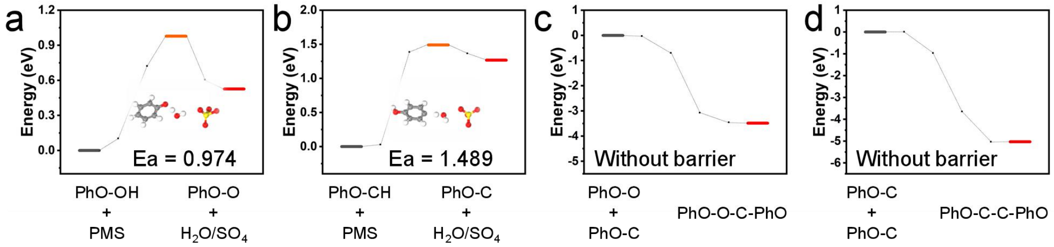

Supplementary Figure 44 | Calculated kinetics of the reaction pathway for PhOH conversion in the control process without the Co_3O_4 surface.

a, PhO-OH + PMS → PhO-O + H_2O/SO_4.

b, PhO-CH + PMS → PhO-C + H_2O/SO_4.

c, PhO-O + PhO-C → PhO-O-C-PhO.

d, PhO-C + PhO-C → PhO-C-C-PhO.

The oxidation of phenol and the production of phenol intermediates could not occur easily since the process is endothermic and due to the high energy barrier. Therefore, the catalyst surface is essential for DOTP reactions to occur.

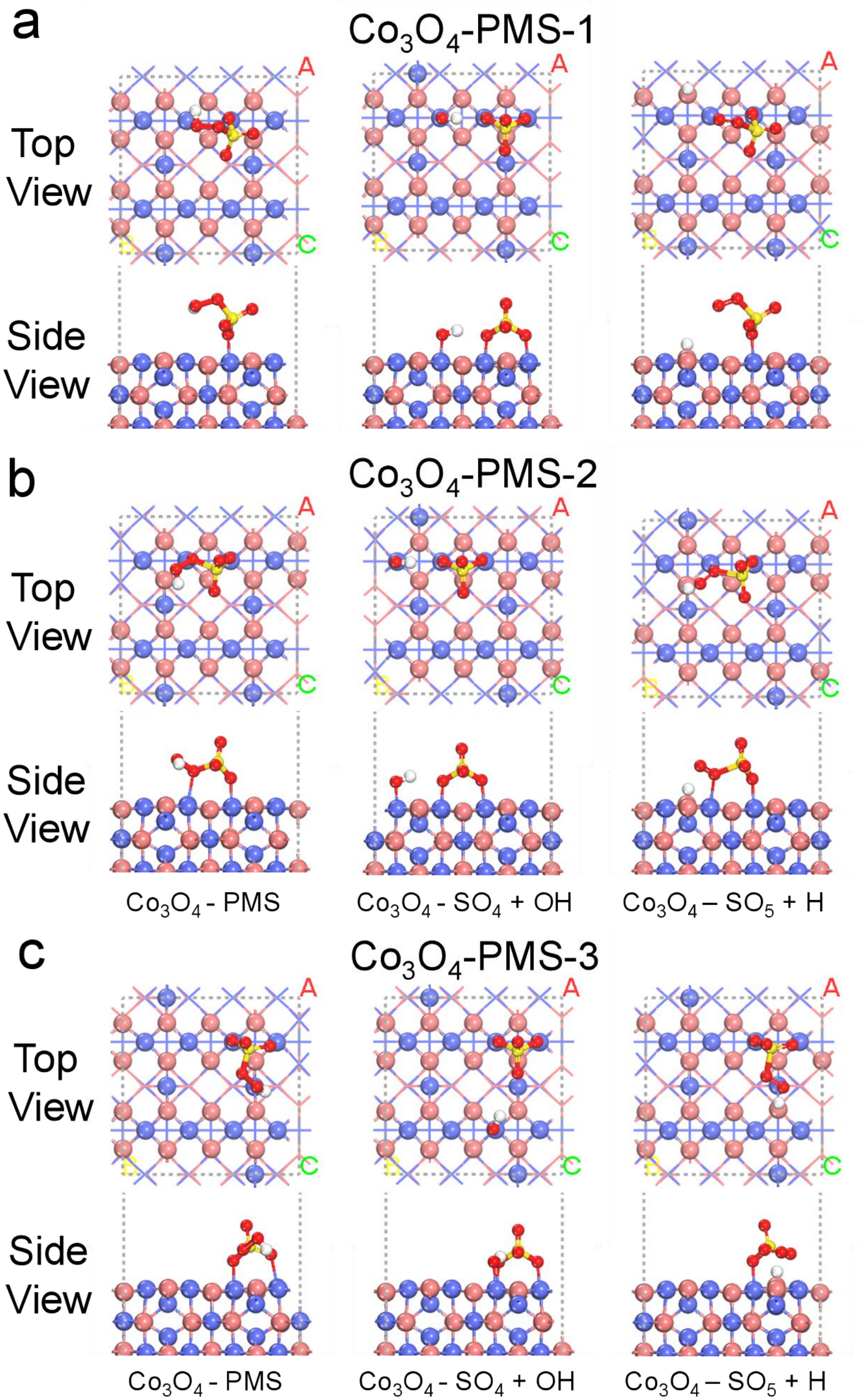
a
Co3O4-PMS-1
Top View
Side View
b
Co3O4-PMS-2
Top View
Side View
Co3O4 - PMS
Co3O4 - SO4 + OH
Co3O4 – SO5 + H
c
Co3O4-PMS-3
Top View
Side View
Co3O4 - PMS
Co3O4 - SO4 + OH
Co3O4 – SO5 + H

Supplementary Figure 45 | Simulation of radical activation process of PMS on Co_3O_4 surface. **a,b,c** Three different adsorption configurations of PMS and their reductive/oxidative reaction products on the Co_3O_4 surface.

In the radical activation of PMS on the catalyst surface, two reactions — reductive reaction and oxidative reaction — of PMS should take place simultaneously to generate radicals continuously by the catalytic cycle [9-11],

The reductive reaction of PMS: $HSO_5^- + e^- \rightarrow SO_4^{\bullet-} + OH^-$

The oxidative reaction of PMS: $HSO_5^- - e^- \rightarrow SO_5^{\bullet-} + H^+$

The thermodynamic free energy of PMS oxidation products is higher than that of PMS itself on the Co_3O_4 surface (Fig. 5**f**). This indicates that the oxidative reaction process does not occur. Therefore, the common view of PMS radical activation on the Co_3O_4 surface in the heterogeneous Fenton-like process is likely to be incorrect, because the oxidative reaction and reductive reaction of PMS could not occur cyclically to continuously produce $SO_4^{\bullet-}/SO_5^{\bullet-}$ radicals.

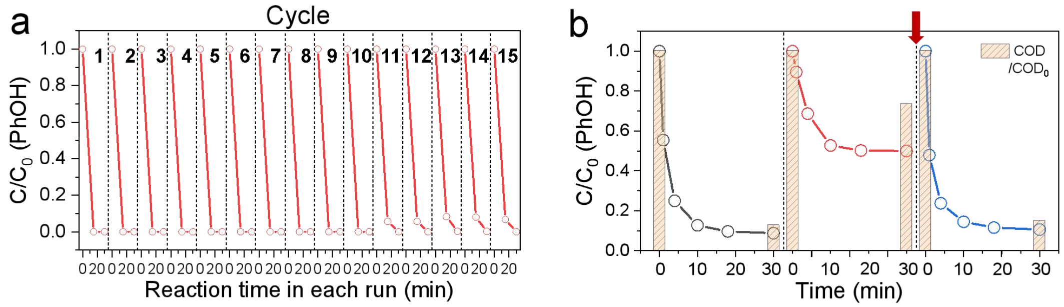

Supplementary Figure 46 | Cycle-test and regeneration performances of Co_3O_4 catalyst for 2,6-MPhOH removal. **a,** Cycle-test performance of Co_3O_4 in the system of low-concentration 2,6-MPhOH. [2,6-MPhOH] = 16 mg/L, [Co_3O_4] = 1.0 g/L, [PMS]:[2,6-MPhOH] = 2 (in molar ratio), pH = 9.0, borate buffer = 20 mM. **b,** Regeneration performance of Co_3O_4 in the system of high-concentration 2,6-MPhOH. [2,6-MPhOH] = 288 mg/L, [Co_3O_4] = 1.0 g/L, [PMS]:[2,6-MPhOH] = 2 (in molar ratio), pH = 9.0, borate buffer = 20 mM.

For the catalytic cycle tests, the used Co_3O_4 was re-dispersed into 2,6-MPhOH solution without drying. For better evaluation of the regeneration effect, a much higher initial concentration of 2,6-MPhOH was used to make the catalyst quickly reach the inactive state (after three cycles). Regeneration process: the used catalyst was washed with a mixed solvent of ethanol and toluene (1:1, v:v) three times (at room temperature for 0.5 h), and then dried at 60 ºC for 12 h.

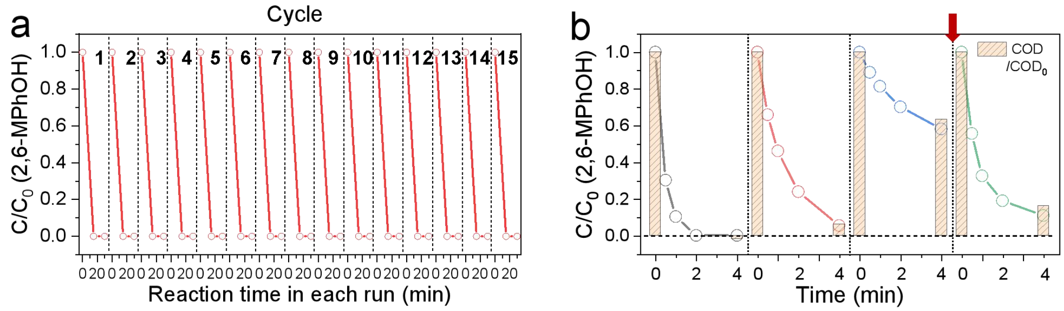

Supplementary Figure 47 | Cycle-test and regeneration performance of Co_3O_4 catalyst for PhOH removal. **a,** Cycle-test performance of Co_3O_4 in the system of low-concentration PhOH. [PhOH] = 12.5 mg/L, [Co_3O_4] = 1.0 g/L, [PMS]:[PhOH] = 2 (in molar ratio), pH = 9.0, borate buffer = 20 mM. **b,** Regeneration performance of Co_3O_4 in the system of high-concentration PhOH. [PhOH] = 250 mg/L, [Co_3O_4] = 1.0 g/L, [PMS]:[PhOH] = 2 (in molar ratio), pH = 9.0, borate buffer = 20 mM.

For the catalytic cycle tests, the used Co_3O_4 was re-dispersed into PhOH solution without drying. For better evaluation of the regeneration effect, a much higher initial concentration of PhOH was used to make the catalyst quickly reach the inactive state (after two cycles). Regeneration process: the used catalyst was annealed at 400 ºC for 1 h in air.

For the pollutants that preferentially undergo the coupling and linear polymerization pathways (e.g., 2,6-MPhOH), their products can be washed off with the mixed solvent of ethanol and toluene to achieve resource recovery (e.g., 3,3',5,5'-tetramethyldiphenoquinone could be washed off by ethanol and used as industrial chemicals) and near-complete restoration of the catalyst activity (Supplementary Figure 36**b**). For the pollutants that mainly undergo a cross-linking polymerization path (e.g., PhOH), a facile annealing treatment can be used to realize the complete regeneration of the catalyst (Supplementary Figure 37**b**).

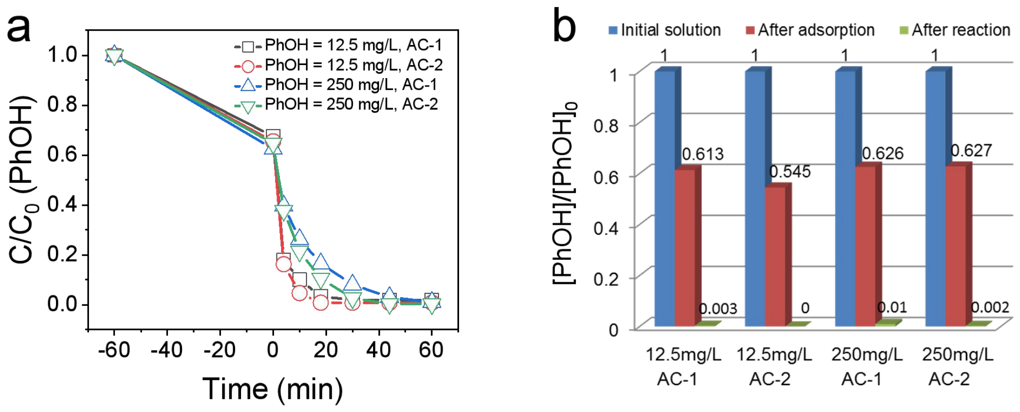

Supplementary Figure 48 | Comparison of PhOH removal (low concentration and high concentration) between adsorption and DOTP reaction using two kinds of activated carbon: the removal process (**a**) and the removal ratio (**b**). Low concentration: [PhOH] = 12.5 mg/L, [AC-1] or [AC-2] = 0.2 g/L; high concentration: [PhOH] = 250 mg/L, [AC-1] or [AC-2] = 1.0 g/L.

Activated carbon acted as the adsorbent in the adsorption system and as the catalyst in the DOTP system. For DOTP, extra PDS was added to the systems for the reaction, [PDS]:[PhOH] = 2:1 (in molar ratio). AC-1, activated carbon purchased from Chemical Reagent Co., China; AC-2, activated carbon purchased from Aladdin Co., China.

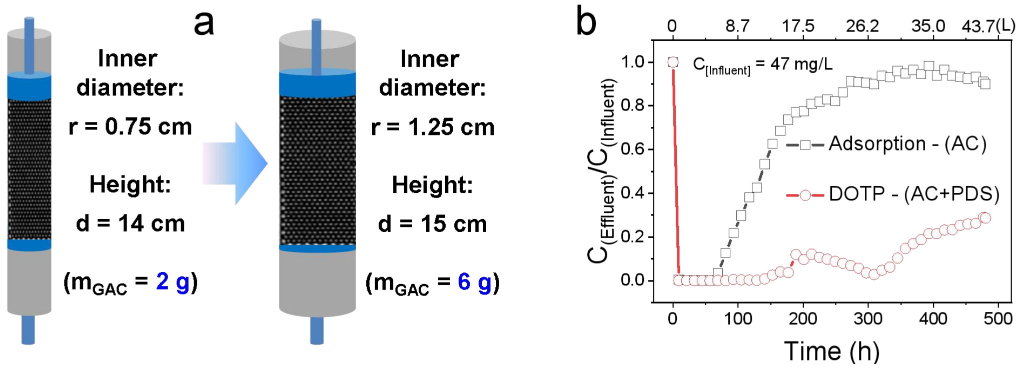

Supplementary Figure 49 | Scale-up effect (three times) of the fixed bed reactor of DOTP and adsorption. a, Enlarged reaction column in the fixed bed filled with 3 times (in weight, 6 g) granular activated carbon than the column in Fig. 7d (the filled mass of GAC was 2 g). **b,** Water purification effect of DOTP and adsorption over 20 days. M_{GAC}, the filled mass of granular activated carbon.

The scale-up experiment results of the fixed bed reactor of DOTP and adsorption corresponded with the result in Fig. 7d, validating the robustness and effectiveness of DOTP for water purification.

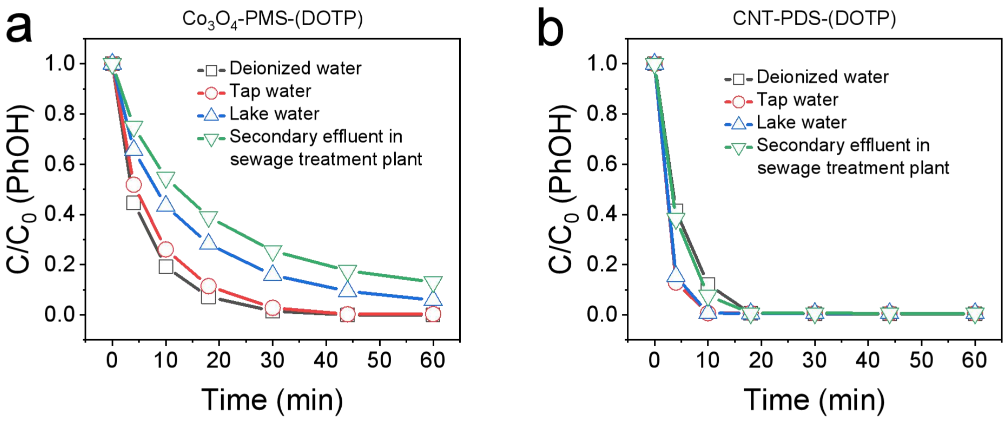

Supplementary Figure 50 | Effects of real environmental water on the DOTP. **a,** Co_3O_4/PMS/PhOH system; **b,** CNT/PDS/PhOH system. [PhOH] = 12.5 mg/L, [Co_3O_4] = 0.2 g/L, [PMS]:[PhOH] = 2 (in molar ratio), pH = 9.0, borate buffer = 20 mM; [PhOH] = 12.5 mg/L, [CNT] = 0.2 g/L, [PDS]:[PhOH] = 2 (in molar ratio). Real environmental water in this work includes tap water, lake water, and secondary effluent from a municipal wastewater treatment plant.

In the Co_3O_4/PMS system, the DOTP was slightly suppressed in the real environmental water, such as lake water and secondary effluent from a municipal wastewater treatment plant. This might be due to the partial covering of the Co_3O_4 surface by real water constituents, such as NOM. While in the CNT/PDS system, the constituents in real water had almost no impact on the DOTP. These effects indicate that the DOTP has a strong anti-interference ability in practical water matrices as no active species participated in the DOTP process (compared to AOPs).

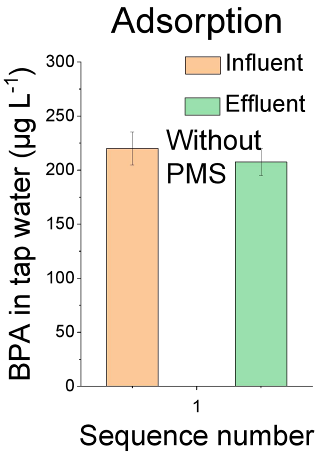

Supplementary Figure 51 | Detected BPA concentrations in the influent and effluent of the membrane catalytic reactor without PMS addition treating 1-L tap water.

The adsorption of BPA on the membrane catalytic reactor (mainly on the Co_3O_4 surface and the filter membrane) was negligible.

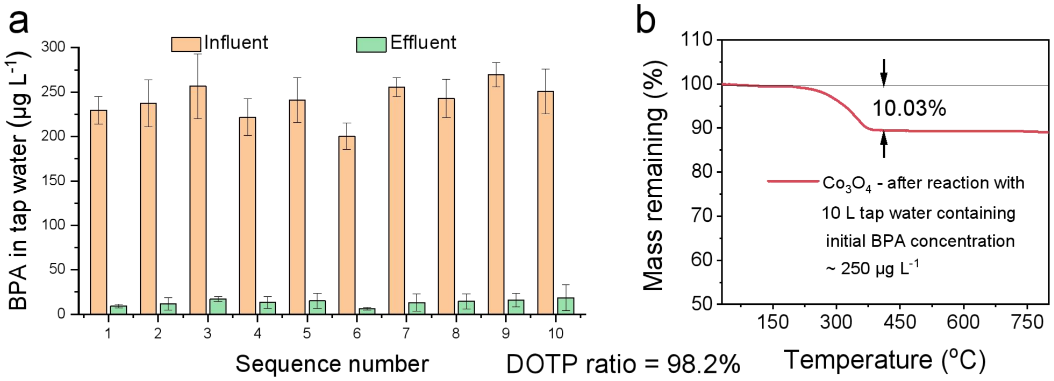

Supplementary Figure 52 | a, Detected BPA concentrations in the influent and effluent for 10 consecutive membrane catalytic reactions (1-L tap water containing initial BPA concentration ~ 250 µg L^{-1} and borate buffer of 2 mM for each reaction). The PMS dosage was slightly increased to 5 times the molar ratio of BPA to enhance the mass transfer and contact rate. **b,** TGA curve of the reacted Co_3O_4 after 10 membrane catalytic reactions shown in **a**. (Mass of Co_3O_4 = 20 mg)

According to the difference in BPA concentrations between the influent and effluent for ten consecutive membrane catalytic reactions, the total removed BPA was 2.27 mg (Fig. S**52a).** The thermogravimetric loss ratio of the Co_3O_4 catalyst after the reaction was 10.03% (Fig. S**52b**), corresponding to 2.23 mg of the surface enriched DOTP products. Therefore, the DOTP ratio was calculated to be 2.23/2.27 = 98.2%.

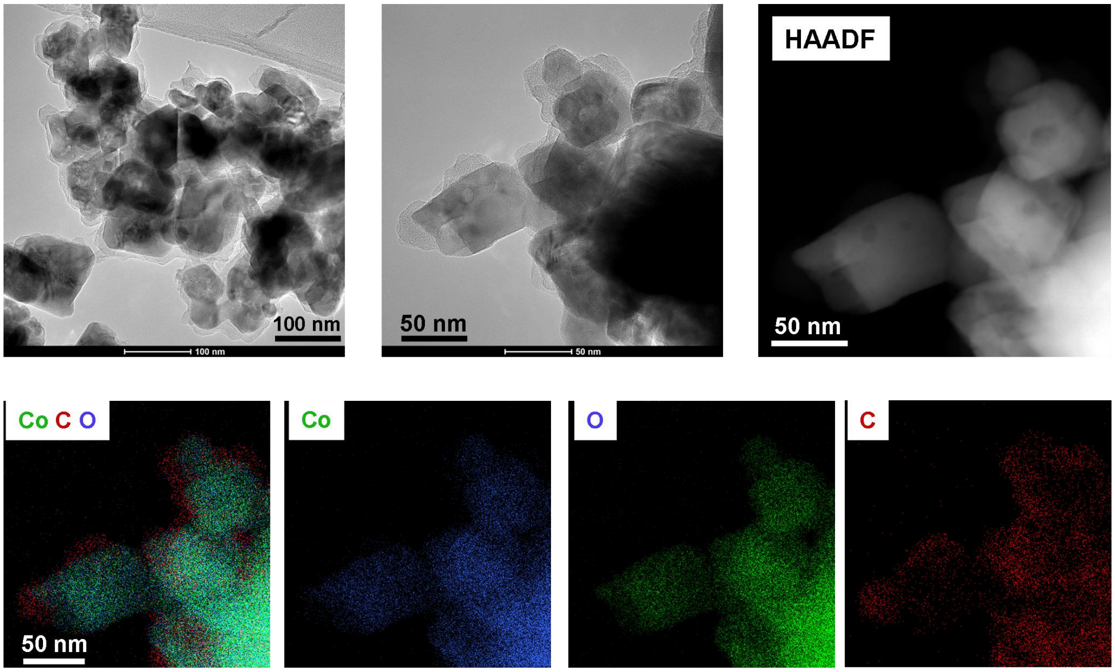

Supplementary Figure 53 | STEM-EDS elemental mapping images of the reacted Co_3O_4 after 10 membrane catalytic reaction runs in Figure R4a.

The thick layer of organic products on the Co_3O_4 surface after 10 membrane catalytic reaction runs corresponded to the DOTP products.

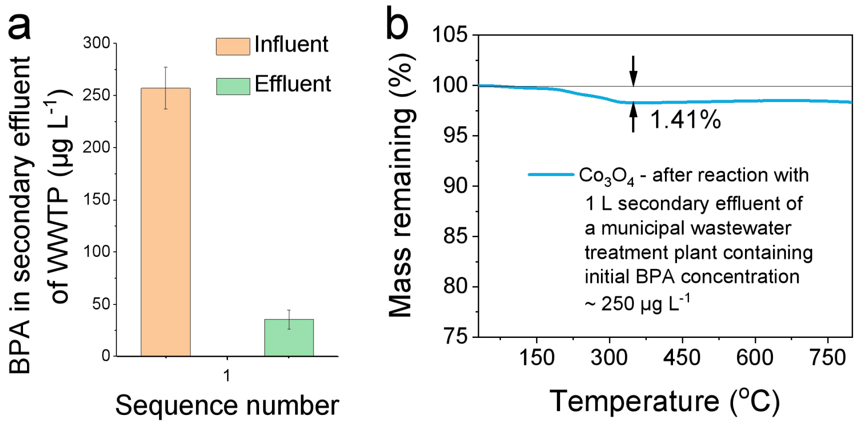

Supplementary Figure 54 | a, Detected BPA concentrations in the influent and effluent of the catalytic membrane reactor for 1-L secondary effluent of a WWTP containing initial BPA concentration of ~250 μg L^{-1} and borate buffer of 2 mM. The PMS dosage was slightly increased to 5 times the molar ratio of BPA to enhance the mass transfer and contact rate. **b,** TGA curve of the reacted Co_3O_4 in **a**. (Mass of Co_3O_4 = 20 mg). Secondary effluent of WWTP, secondary effluent of a municipal wastewater treatment plant, Hefei, China.

Since the secondary effluent of the WWTP contains dissolved organic matter (DOM) and other components that cause a large membrane filtration resistance, we conducted one round of the membrane catalytic reaction only (i.e., the solution volume was 1 L). As shown in Fig. S**54a**, 85% of BPA was removed through the single round of treatment, and obvious thermogravimetric loss (1.41%) was observed on the catalyst after the reaction. The calculated DOTP ratio (140%) exceeds 100%, which might be owning to the presence of some reactive substances in DOM. Nevertheless, the trend of the decomposition temperature of the organic products (~300 ºC) proves the formation of obvious DOTP products.

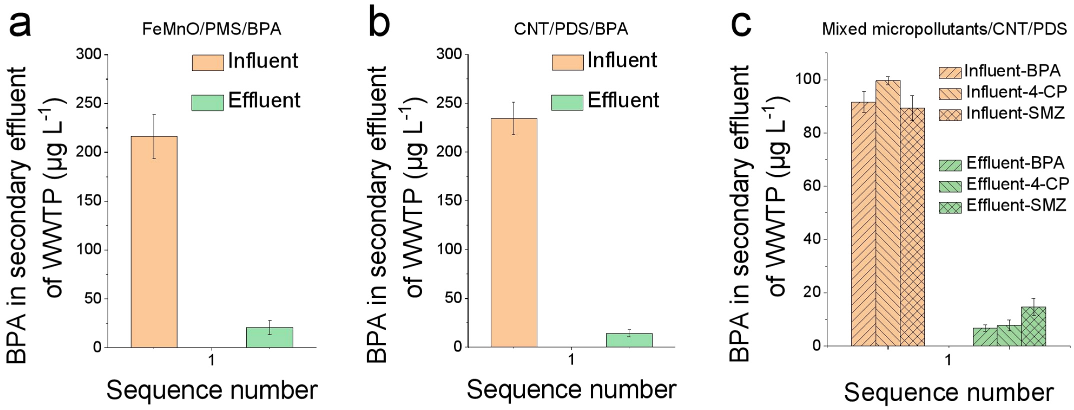

Supplementary Figure 55 | a, b, Detected BPA concentrations in the influent and effluent for 1-L secondary effluent of WWTP by using (**a**) FeMnO and (**b**) CNT as the membrane catalysts. **c,** Detected BPA, 4-CP, and SMZ concentrations in the influent and effluent for 1-L secondary effluent of WWTP containing a mixture of micropollutants (BPA, 4-CP, and SMZ) (catalyst mass = 20 mg, PMS or PDS dosage = 5 times that of the total pollutants). 4-CP, 4-chlorophenol; SMZ, sulfamethoxazole.

Supplementary Table 1 | Summary of the electron-equivalent non-conservation data according to the literature

Catalyst	Pollutant concentration (mM) [a]	TOC removal (%) [b]	**e^- equivalence of reductant (mM) [c]**	Oxidant concentration (mM) [d]	**e^- equivalence of oxidant (mM) [e]**	Ref.
Co_3O_4	[4-CP] = 0.780	72.2	**14.642**	[PMS] = 0.800	**1.600**	[12]
FeMnO	[BPA] = 0.044	80.0	**2.534**	[PMS] = 0.651	**1.302**	[10]
MnOx	[4-CP] = 0.623	81.0	**13.120**	[PMS] = 1.500	**3.000**	[13]
CuO/Fe_3O_4	[BPA] = 0.088	97.0	**6.146**	[PMS] = 0.300	**0.600**	[14]
CNT	[2,4-DCP] = 0.050	76.3	**0.916**	[PDS] = 0.050	**0.100**	[15]
Biochar	[BPA] = 0.044	80.0	**2.534**	[PMS] = 0.326	**0.652**	[16]

[a, b, d] These values were obtained from the corresponding literature.

[c] This value was calculated according to the amount of pollutant mineralization to CO_2 and H_2O. For the Co_3O_4/PMS/4-CP system as an example, 1 mol 4-CP → 6 mol CO_2 + 2 mol H_2O + 1 mol HCl + 26 mol e^-; the e^- equivalence of the reductant was calculated from: [pollutant]×(TOC removal) ×26 = 0.780×0.722×26 = 14.642.

[e] This value was calculated according to the actual dosage of oxidant (PMS or PDS). One mole of PMS or PDS accepts two moles of electrons in the reaction process. For Co_3O_4/PMS/4-CP system as an example, the e^- equivalence of oxidant was calculated from: [PMS or PDS] ×2 = 0.800×2 = 1.600.

In AOP (removed total organic carbon (TOC) via degradation/mineralization process), the e^- equivalence of the oxidant (i.e., the obtained electron equivalent of PMS or PDS) should be greater than or equal to that of the reductant (i.e., the given electron equivalent of pollutant). But the results in Table S1 show that the obtained electron equivalent calculated from the actual dosage of PMS or PDS was much less than the given electron equivalent calculated from the TOC removal (via AOP), indicating a contradiction of electron equivalent non-conservation. This contradiction of electron equivalent non-conservation is universal in the studies concerning persulfate-based heterogeneous Fenton-like reactions, in which the pollutant removal processes have long been recognized as AOPs by mistake.

Supplementary Table 2 | Calculated energy in reaction pathway of PhOH with and without the Co_3O_4 surface

Structure	Energy (eV)	Structure	Energy (eV)	ΔE (eV)
Co_3O_4 + PhO-OH + PMS	-854.17	Co_3O_4 + PhO-O + H_2O/SO_4	-856.29	-2.12
Co_3O_4 + PhO-CH + PMS	-854.62	Co_3O_4 + PhO-C + H_2O/SO_4	-856.28	-1.66
Co_3O_4 + PhO-O + PhO-C	-890.17	Co_3O_4 + PhO-O-C-PhO	-890.44	-0.27
Co_3O_4 + PhO-C + PhO-C	-889.79	Co_3O_4 + PhO-C-C-PhO	-891.15	-1.36
PhO-OH + PMS	-119.96	PhO-O + H_2O/SO_4	-119.43	0.53
PhO-CH + PMS	-119.54	PhO-C + H_2O/SO_4	-118.28	1.26
PhO-O + PhO-C	-154.42	PhO-O-C-PhO	-157.90	-3.48
PhO-C + PhO-C	-153.35	PhO-C-C-PhO	-158.37	-5.02

Supplementary Table 3 | DOTP ratio of granular activated carbon in the continuous-flow operation (Fig. 7d and e) over 80% (the DOTP ratio of the continuous-flow operation = the accumulated mass / the total mass of pollutant flowed through the column = 0.54 g/(0.047 g/L*14 L) = 82%)

	Adsorption	DOTP
The mass before operation (g) in Fig.7d and e	2.00	2.00
The mass after operation (g) in Fig.7d and e	2.12	2.54
Capacity (g/g)	0.06	0.27

Supplementary Table 4 | Cost of reagents used in DOTPs, AOPs, and adsorption processes

	Cost ($/kg)
Hydrogen peroxide (H_2O_2)	1.5 [a]
Peroxymonosulfate (PMS)	2.2 [a]
Peroxydisulfate (PDS)	0.74 [a]
Commercial Activated carbon	2 ~ 47 [b]

[a] Prices obtained from references [17-19].

[b] Prices obtained from reference [20].

Supplementary Table 5 | Estimated costs for DOTP, adsorption, and AOP

Treatment of 1 kg PhOH	DOTP	Adsorption	AOP
Solids consumption (Kg) (catalysts or adsorbents)	~ 3.7 [a]	~ 16.7 [b]	> 0 [c]
Oxidants consumption (Kg)	5.7 [d]	0	287.2 [e]
Cost ($) [f]	11.6	33.4	> 212.4

[a] The adsorption capacity of granular activated carbon was 0.06 g/g, according to Table S3.

[b] The DOTP decontamination capacity of granular activated carbon was 0.27 g/g, according to table S3.

[c] The dosage of catalyst used in AOPs was not straightforward to calculate, but it is estimated to be greater than 0.

[d] The molar amount of oxidizing agent consumed in DOTPs was less than twice the molar amount of pollutant.

[e] The molar amount of oxidizing agent consumed in AOPs was larger than 100 times the molar amount of pollutant, according to Fig. 7c.

[f] The costs of activated carbon and oxidizing agent were calculated at the lowest prices, as listed in Table S4.

(M_{PhOH} = 94 g/mol, M_{PDS} = 270 g/mol, $Price_{PDS}$ = 0.74 $/Kg, $Price_{activated\ carbon}$ = 2 $/Kg)

Supplementary Table 6 | Determined Co^{2+} leaching amount for the Co_3O_4/PMS/PhOH reaction systems at different pH values by ICP-MS

pH	3.8	7.8	9.1	10.0
Co^{2+} leaching (mg L^{-1})	0.359	0.005	0.003	0

Supplementary Note 1

Metallic oxides and carbonaceous materials are the most widely used heterogeneous catalysts in the persulfate-based Fenton-like systems[10,21-23]. These two kinds of catalysts are promising for practical applications due to their low costs. In this study, we employed Co_3O_4, FeMnO, and CuO_x as the representative metallic oxides, and CNT and biochar as the representative carbonaceous materials. These catalysts have been commonly reported for PMS activation in previous works involving the degradation/mineralization process via the radical mechanism, singlet oxygen mechanism, etc. The nanocatalysts used in a DOTP should not contain reductive metals, such as zero-valent iron (ZVI) or materials loaded with ZVI (ZVI@C). Strictly speaking, these reductive solids (e.g., zero-valent metals) are not catalysts, and they will be oxidized to metal cations and then released into the reaction solution to induce conventional radical-based AOP.

Supplementary Note 2

The DOTP reaction occurs between oxidants and pollutants on the catalyst surface. In-situ EPR testing is accomplished by adding DMPO to the catalysts + oxidants + pollutants system (the real reaction system with pollutant participating) and tested time-dependently for detection of •OH and $SO_4^{\bullet-}$. If the pollutant removal process involves radicals, then the radicals signal should be detected in the in-situ EPR spectra, such as in the typical •OH system (Fe^{2+}/H_2O_2/PhOH) and $SO_4^{\bullet-}$ system (ZVI/PMS/PhOH) (Fig. **4h** and Fig. S**33**). However, in the Co_3O_4/PMS/PhOH system, there were no radical signals (i.e., DMPO-OH and DMPO-SO_4), but the signal for DMPO-X was observed in the EPR spectra from the beginning of the reaction and it then went through a process of initial increase and later decrease (Fig. **4g** and Figs. S**31b**-S**32**). Combined with the radical quantitation fluorescence detection (Fig. **4i** and Fig. S**34**) and scavenging (Fig. S**35**) results that no radicals were involved, it can be concluded that the DMPO-X might be the product of the direct transfer of an oxygen atom (direct $2e^-$ oxidation) from PMS to

DMPO (DMPO, 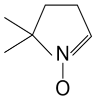 ; DMPO-X, 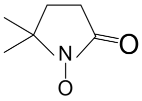) on the Co_3O_4 surface [24]. Since DMPO could not be oxidized by PMS in the absence of Co_3O_4 (Fig. S**31a**), the direct oxygen transfer reaction should occur after the activation of DMPO and PMS by the catalyst surface.

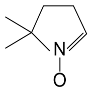 + PMS ($HO\text{-}O\text{-}SO_3^-$) $\xrightarrow{\text{nanocatalyst surface}}$ 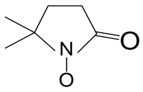 + SO_4^{2-} + H^+

Supplementary Note 3

In the field of polymer synthesis, the polymerization reaction of phenols using a copper-ammonia complex as the catalyst and O_2 as the oxidant is commonly employed to synthesize polyphenylene ether (PPO) [25,26].

phenol + $1/2O_2$ $\xrightarrow{\text{catalyst (copper ammonia complex)}}$ PPO and coupling byproducts

PPO is one of the top five engineering plastics in the world, and its reaction mechanism has been studied extensively. The reaction involves a one-step two electron redox process[27-29]. Following this mechanism (the $2e^-$ redox reaction of O_2 and phenol), the generated phenoxonium intermediates, stabilized by the copper ammonia complex, polymerize with other phenols to form PPO (with a small amount of side-reaction). Based on the reaction condition analysis (Fig. **1**), the product analysis (Fig. **3**), the QSAR analysis (Table **1**), and the mechanism analysis (Fig. **4**), the DOTP for pollutant removal is analogous to this process, but occurs on the heterogeneous nanocatalyst surface.

phenol + PMS $\xrightarrow{\text{nanocatalyst surface}}$ polymerization products (PPO) and coupling products (biphenyl quinone)

The heterogeneous catalyst surface can also play a role in stabilizing the phenoxonium intermediates.

Supplementary Note 4

The energy barriers of the PMS/PhOH reaction decreased from greater than 0.974 eV to less than 0.415 eV without and with the Co_3O_4 surface (Figs. S**32** and S**34**), indicating the activation function of catalyst surface for PMS (i.e., oxidant) and PhOH (i.e., reductant) molecules (Fig. S**40**). The thermodynamic free energy of the PhOH oxidative intermediates (relative to reactants) decreased from 1.27 and 0.53 eV to -1.66 and -2.12 eV without and with the Co_3O_4 surface, respectively (Fig. **5e**), indicating the stabilization function of catalyst surface. The thermodynamic free energy of the polymerization product and coupling product (relative to reactants) decreased from -2.49 and -1.69 eV to -4.68 and -4.05 eV without and with the Co_3O_4 surface, respectively (Fig. **5e**), indicating the accumulation function of catalyst surface. The lower thermodynamic free energy corresponds to the more stable of the molecule state.

Supplementary Note 5

According to the chemometrics of AOPs, complete mineralization of each organic molecule typically involves the transfer of tens of electrons from the pollutant molecule to the oxidant (e.g., 28 electrons for one phenol molecule), which requires the addition of large amounts of oxidizing agents and/or energy. As shown in Fig. **7a** and **7b**, the TOC residual was very high in the AOP systems (e.g., Fe^{2+}/H_2O_2 and ZVI/PMS) with a low dosage of oxidant (4 times the molar amount of PhOH), even though most of the PhOH was removed. When the dosage of the oxidant was increased to 100 times the molar amount of pollutant, the TOC removal still did not exceed 53% (Fig. **7c**). To achieve higher TOC removal efficiency, the dosage of oxidizing agents is usually two to three orders of magnitude above the molar amount of pollutants [19,30,31]. However, DOTPs require the transfer of only 2 to 4 electrons (e.g., 2,6-M-PhOH and 4-CP, 2 electrons; PhOH and BPA, 4 electrons) from the pollutant molecule to the oxidant in order to accomplish complete contaminant removal from water. Thus, the amount of oxidizing agent can be substantially reduced in DOTPs.

Supplementary Note 6

DOTPs demonstrate significant advantages over conventional adsorption processes, even though both processes involve water purification via pollutant transfer from water to a solid surface. Unlike adsorption processes, which usually suffer from incomplete pollutant removal due to sluggish kinetics and a low adsorption-desorption equilibrium constant (i.e., pollutants distribute in the aqueous phase and on the solid surface in a certain proportion)[20], DOTPs can completely remove organic pollutants from water (nearly 100%). Using two types of activated carbon as an example (Supplementary Figure **39**), a simple adsorption process removed less than half of the PhOH in the initial solution; however, DOTP achieved near complete removal of PhOH within the same time period since the removal process consisted of chemical accumulation (i.e., oxidative coupling and polymerization reaction) rather than physical adsorption. The advantages were also verified by the continuous-flow operation in a fixed bed reactor (Fig. **7d**). For the same mass of activated carbon as catalyst, the DOTP could continue to remove phenol effectively 4.5 times longer than the adsorption process (Fig. **7e** and table S**3**). The scale-up of the fixed bed reactor further verified the robustness of DOTP (Supplementary Figure **40**).

Supplementary References

1 Liang, C. J., Huang, C. F., Mohanty, N. & Kurakalva, R. M. A rapid spectrophotometric determination of persulfate anion in ISCO. Chemosphere 73, 1540-1543 (2008).

2 National Institute of Advanced Industrial Science and Technology (AIST), Japan. https://sdbs.db.aist.go.jp/sdbs/cgi-bin/direct_frame_top.cgi.

3 Huang, K. Z. & Zhang, H. C. Direct Electron-Transfer-Based Peroxymonosulfate Activation by Iron-Doped Manganese Oxide (delta-MnO_2) and the Development of Galvanic Oxidation Processes (GOPs). Environ Sci Technol 53, 12610-12620 (2019).

4 Liu, H. L., Hou, P., Zhang, W. X., Kim, Y. K. & Wu, J. The synthesis and characterization of polymer-coated FeAu multifunctional nanoparticles. Nanotechnology 21 (2010).

5 Gomez-Gallego, M. & Sierra, M. A. Kinetic Isotope Effects in the Study of Organometallic Reaction Mechanisms. Chem Rev 111, 4857-4963 (2011).

6 Gao, X. L., Wang, P., Zeng, L., Tang, S. & Lei, A. W. Cobalt(II)-Catalyzed Electrooxidative C-H Amination of Arenes with Alkylamines. J Am Chem Soc 140, 4195-4199 (2018).

7 Niu, L. B. et al. Photo-induced oxidant-free oxidative C-H/N-H cross-coupling between arenes and azoles. Nature Communications 8 (2017).

8 Wang, Z., Ni, J. Z., Kuninobu, Y. & Kanai, M. Copper-Catalyzed Intramolecular C(sp(3))-H and C(sp(2))-H Amidation by Oxidative Cyclization. Angew Chem Int Edit 53, 3496-3499 (2014).

9 Kamagate, M., Pasturel, M., Brigante, M. & Hanna, K. Mineralization Enhancement of Pharmaceutical Contaminants by Radical-Based Oxidation Promoted by Oxide-Bound Metal Ions. Environ Sci Technol 54, 476-485 (2020).

10 Huang, G. X., Wang, C. Y., Yang, C. W., Guo, P. C. & Yu, H. Q. Degradation of Bisphenol A by Peroxymonosulfate Catalytically Activated with $Mn_{1.8}Fe_{1.2}O_4$ Nanospheres: Synergism between Mn and Fe. Environ Sci Technol 51, 12611-12618 (2017).

11 Oh, W. D., Dong, Z. L. & Lim, T. T. Generation of Sulfate Radical through Heterogeneous Catalysis for Organic Contaminants Removal: Current Development, Challenges and Prospects. Appl Catal B-Environ 194, 169-201 (2016).

12 Zeng, T., Zhang, X. L., Wang, S. H., Niu, H. Y. & Cai, Y. Q. Spatial Confinement of

a Co_3O_4 Catalyst in Hollow Metal-Organic Frameworks as a Nanoreactor for Improved Degradation of Organic Pollutants. Environ Sci Technol 49, 2350-2357 (2015).

13 Khan, A. et al. Facile synthesis of yolk shell Mn_2O_3@$Mn5O_8$ as an effective catalyst for peroxymonosulfate activation. Phys Chem Chem Phys 20, 13909-13919 (2018).

14 Ding, Y. B. et al. Deep mineralization of bisphenol A by catalytic peroxymonosulfate activation with nano CuO/Fe_3O_4 with strong Cu-Fe interaction. Chem Eng J 384, 123378 (2020).

15 Cheng, X., Guo, H. G., Zhang, Y. L., Korshin, G. V. & Yang, B. Insights into the mechanism of nonradical reactions of persulfate activated by carbon nanotubes: Activation performance and structure-function relationship. Water Res 157, 406-414 (2019).

16 Huang, B. C., Jiang, J., Huang, G. X. & Yu, H. Q. Sludge biochar-based catalysts for improved pollutant degradation by activating peroxymonosulfate. J Mater Chem A 6, 8978-8985 (2018).

17 Zhang, T. et al. Efficient Peroxydisulfate Activation Process Not Relying on Sulfate Radical Generation for Water Pollutant Degradation. Environ Sci Technol 48, 5868-5875 (2014).

18 Lau, T. K., Chu, W. & Graham, N. J. D. The aqueous degradation of butylated hydroxyanisole by $UV/S_2O_8^{2-}$: Study of reaction mechanisms via dimerization and mineralization. Environ Sci Technol 41, 613-619 (2007).

19 Anipsitakis, G. P. & Dionysiou, D. D. Degradation of organic contaminants in water with sulfate radicals generated by the conjunction of peroxymonosulfate with cobalt. Environ Sci Technol 37, 4790-4797 (2003).

20 Alsbaiee, A. et al. Rapid removal of organic micropollutants from water by a porous beta-cyclodextrin polymer. Nature 529, 190-194 (2016).

21 Lee, J., von Gunten, U. & Kim, J. H. Persulfate-Based Advanced Oxidation: Critical Assessment of Opportunities and Roadblocks. Environ Sci Technol 54, 3064-3081 (2020).

22 Yun, E. T., Lee, J. H., Kim, J., Park, H. D. & Lee, J. Identifying the Nonradical Mechanism in the Peroxymonosulfate Activation Process: Singlet Oxygenation Versus Mediated Electron Transfer. Environ Sci Technol 52, 7032-7042 (2018).

23 Hu, P. D. & Long, M. C. Cobalt-catalyzed sulfate radical-based advanced oxidation: A review on heterogeneous catalysts and applications. Appl Catal B-Environ 181, 103-117 (2016).

24 Stan, S. D. & Daeschel, M. A. 5,5-Dimethyl-2-pyrrolidone-N-oxyl formation in

electron spin resonance studies of electrolyzed NaCl solution using 5,5-dimethyl-1-pyrroline-N-oxide as a spin trapping agent. J Agr Food Chem 53, 4906-4910 (2005).

25 Chen, C. W., Lin, I. H., Lin, C. C., Lin, J. L. & Horie, M. Synthesis of poly(2,6-dimethy1-1,4-phenylene oxide) derivatives in water using water-soluble copper complex catalyst with natural ligands. Polymer 54, 5684-5690 (2013).

26 Hay, A. S. Polymerization by Oxidative Coupling .2. Oxidation of 2,6-Disubstituted Phenols. J Polym Sci 58, 581-& (1962).

27 Zhao, Y., Wu, L. B., Li, B. G. & Zhu, S. P. The Effect of Ligand Molecular Weight on Copper Salt Catalyzed Oxidative Coupling Polymerization of 2,6-Dimethylphenol. J Appl Polym Sci 117, 3473-3481 (2010).

28 Gupta, S., van Dijk, J. A. P. P., Gamez, P., Challa, G. & Reedijk, J. Mechanistic studies for the polymerization of 2,6-dimethylphenol to poly(2,6-dimethyl-1,4-phenylene ether): LC-MS analyses showing rearrangement and redistribution products. Appl Catal a-Gen 319, 163-170 (2007).

29 Baesjou, P. J., Driessen, W. L., Challa, G. & Reedijk, J. Ab initio calculations on 2,6-dimethylphenol and 4-(2,6-dimethylphenoxy)-2,6-dimethylphenol. Evidence of an important role for the phenoxonium cation in the copper-catalyzed oxidative phenol coupling reaction. J Am Chem Soc 119, 12590-12594 (1997).

30 Lyu, L., Zhang, L. L., Wang, Q. Y., Nie, Y. L. & Hu, C. Enhanced Fenton Catalytic Efficiency of gamma-Cu-Al_2O_3 by sigma-Cu^{2+}-Ligand Complexes from Aromatic Pollutant Degradation. Environ Sci Technol 49, 8639-8647 (2015).

31 Sen Gupta, S. et al. Rapid total destruction of chlorophenols by activated hydrogen peroxide. Science 296, 326-328 (2002).
